# Evaluation of the efficacy of an internet-based pain education and exercise program for chronic musculoskeletal pain in comparison with online self-management booklet: a protocol of a randomised controlled trial with assessor-blinded, 12-month follow-up, and economic evaluation

**DOI:** 10.1186/s12891-020-03423-x

**Published:** 2020-06-26

**Authors:** Iuri Fioratti, Bruno T. Saragiotto, Felipe J. J. Reis, Gisela C. Miyamoto, Hopin Lee, Tiê P. Yamato, Junior V. Fandim, Blake Dear, Chris G. Maher, Leonardo O. P. Costa

**Affiliations:** 1 Masters and Doctoral Programs in Physical Therapy, Universidade Cidade de São Paulo, Rua Cesário Galeno, 448/475, Tatuape, São Paulo, 03071-000 Brazil; 2 Centre for Pain, Health, and Lifestyle Brazil, Sao Paulo, Brazil; 3 Institute for Musculoskeletal Health, The University of Sydney and Sydney Local Health District, Sydney, Australia; 4 Department of Physical Therapy, Instituto Federal do Rio de Janeiro, Rio de Janeiro, Brazil; 5 Centre for Statistics in Medicine, Rehabilitation Research in Oxford, Nuffield Department of Orthopaedics Rheumatology and Musculoskeletal Sciences (NDORMS), University of Oxford, Oxford, UK; 6 School of Medicine and Public Health, University of Newcastle, Newcastle, New South Wales Australia; 7 Department of Psychology, Macquarie University, Sydney, Australia; 8 Sydney School of Public Health, The University of Sydney, Sydney, Australia

**Keywords:** Chronic pain, Chronic musculoskeletal pain, Internet-based, Cost-effectiveness analysis, Study protocol

## Abstract

**Background:**

Chronic musculoskeletal pain is one of the main causes of years lived with disability and generates the highest cost of health care among chronic pain conditions. Internet-based treatments have been shown to be an alternative for the treatment of musculoskeletal conditions, in addition to reducing barriers such as travel, high demands on the public health system, lack of time, lack of insurance coverage for private care, and high costs for long-term treatment. The aim of this clinical trial is to develop and test the effectiveness and cost-effectiveness of, an internet-based self-management program based on pain education and exercise for people with chronic musculoskeletal pain.

**Methods:**

This is a prospectively registered, assessor-blinded, two-arm randomised controlled trial with economic evaluation comparing the Internet-based pain education and exercise intervention with a control group that will receive an online booklet. One hundred and sixty patients will be recruited from Sao Paulo, Brazil. Follow-ups will be conducted in post-treatment, 6 and 12 months after randomisation. The conduct of the study, as well as the evaluations and follow-ups will be carried out entirely remotely, through online platforms and telephone calls. The primary outcome will be pain intensity at post-treatment (8 weeks) measured using the 11-item Pain Numerical Rating Scale. Secondary outcomes will be biopsychosocial factors presents in the chronic musculoskeletal pain condition. Costs due to chronic musculoskeletal pain will be also measured, and cost-effectiveness analysis from a societal perspective will performed.

**Discussion:**

Our hypothesis is that internet-based pain education and exercise will be better than an online booklet in reducing pain and improving biopsychosocial outcomes in patients with chronic musculoskeletal pain. In addition, we believe that there will be good acceptance of patients for the internet-based intervention and that internet-based intervention will be more cost effective than the online booklet.

**Trial registration:**

The study was prospectively registered at ClinicalTrials.gov (NCT04274439, registered 18 February 2020).

## Background

Chronic pain is a major burden on the individual and society [1]. It is estimated that about one third of the adult population worldwide suffer from chronic pain, with higher prevalence rates reported for low-income countries [2–5]. In Brazil, the prevalence of chronic pain is around 39% [6] in the adult population, and up to 61% in adult workers [7]. Chronic pain has also been reported as the main reason for people seeking care in outpatient clinics in Brazil [6].

The most common conditions responsible for chronic pain are musculoskeletal disorders of the back, neck and upper limbs; as well as knee and hip osteoarthritis. In the most recent Global Burden of Disease Study, musculoskeletal pain accounted for more than 20% of years lived with disability in the population, and low back pain alone was the leading cause of years lived with disability across the globe [8]. Musculoskeletal pain is responsible for the highest overall healthcare costs of all chronic pain conditions, with most of the costs related to the use of outpatient services [9].

Treatment for chronic musculoskeletal pain is a challenge for clinicians since it is often accompanied by many factors that are associated with pain, such as disability, emotional distress, work absenteeism, reduced quality of life, and even early mortality [10–14]. Therefore, it has been suggested that a multimodal treatment approach focusing on pain management that incorporates both physical and psychosocial factors may address better symptoms of patients with chronic musculoskeletal pain [15–18].

Internet-based self-management programs have grown with the development of new technologies and have been extensively used for delivering healthcare in many areas [19–22]. Internet-based self-management programs overcomes some of the potential barriers that patients face with traditional face-to-face healthcare, such as travel (distance, traffic, transport, and time consumed), high demand of the public health system (e.g. long waiting lists), lack of time, lack of insurance coverage for private care, and high costs for long-term treatment. Previous studies have shown that internet-based self-management programs may be effective for reducing pain and disability in patients with chronic pain [23, 24]. However, there is still little research on the development and testing of remotely-delivered programs for the management of chronic musculoskeletal pain, especially in low- and middle-income countries.

The aim of this clinical trial is to develop and test the effectiveness and cost-effectiveness of an internet-based self-management program based on pain education and exercise for people with chronic musculoskeletal pain.

## Methods

### Elaboration protocol

This study protocol follows the recommendations of the SPIRIT Statement [25] for clinical trial protocols elaboration and is reported according to the CONSORT Statement [26].

### Study design

This is a prospectively registered, assessor-blinded, two-arm randomised controlled trial with economic evaluation.

### Settings and eligibility criteria

Patients will be recruited from the community in Brazil. We will include patients aged between 18 and 60 years, seeking treatment for any chronic musculoskeletal pain condition. Eligibility criteria are: pain of at least 3 points on a 0 to 10 Pain Numerical Rating Scale, able to read and understand Portuguese and with internet access. Chronic pain will be defined as pain lasting more than 12 weeks [27].

Exclusion criteria are: nerve root compromise, serious pathologies (e.g., fracture, tumor, inflammatory, autoimmune, and infectious diseases), serious cardiovascular and metabolic diseases (e.g., coronary heart disease, cardiac insufficiency, decompensated diabetes), recent orthopaedic surgery (over the last 12 months), scheduled to undergo surgery in the next six months, or pregnancy. Patients will also be excluded if there is any contraindication to exercise. We will pre-screen for physical activity participation at baseline using the Physical Activity Readiness Questionnaire (PAR-Q) Portuguese version [28, 29].

### Procedure

**Fig. 1:**
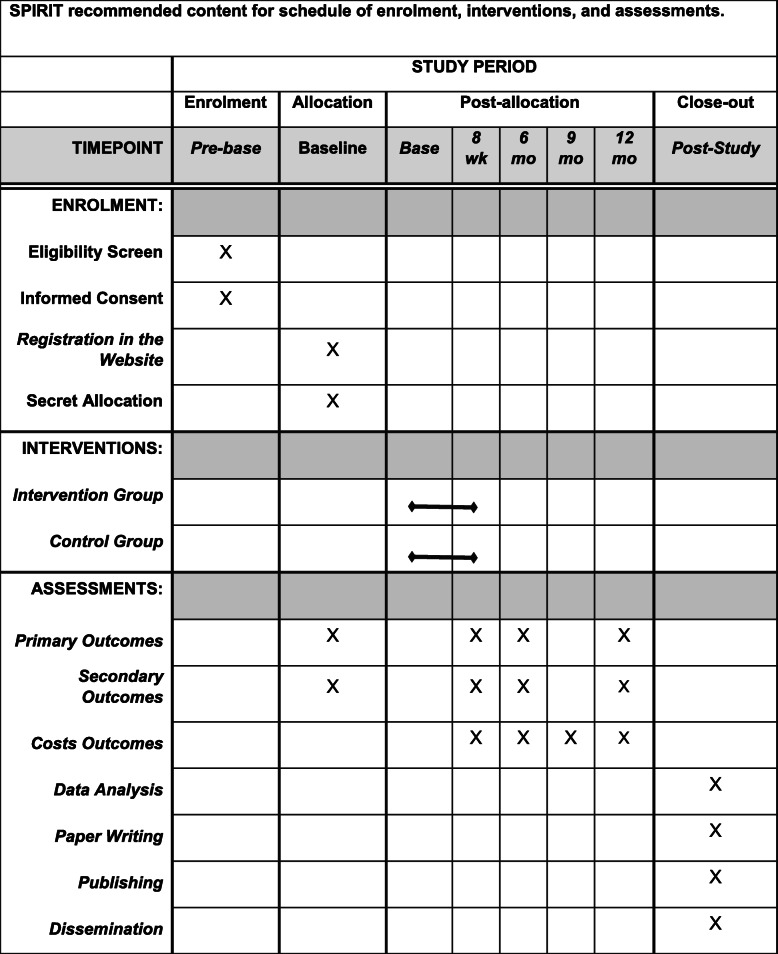
SPIRIT recommended content for schedule of enrolment, interventions, and assessments

The blinded assessor will screen potential participants to determine eligibility. Eligible patients will receive the online consent form and will be informed about the aims of the study. The blinded assessor will then collect the patient’s sociodemographic data, medical history, and the data related to the study outcomes for baseline assessment. The baseline assessment will be performed via videoconference using a personalised link using Whereby platform. All other assessments will be collected by telephone calls, smartphone messages or e-mail (8 weeks, 6, and 12 months after randomisation) through a questionnaire created on the Typeform platform (Fig. 1). All data entry will be coded and double-entered into a Microsoft Excel spreadsheet. A second blinded researcher will double-check the data prior to the analysis.

### Outcome measures

The primary outcome will be pain intensity at 8 weeks measured using the Pain Numerical Rating Scale [30], a numerical scale where 0 indicates no pain and 10 indicates maximum pain intensity.

The secondary outcomes will be as follows:

- Pain intensity at 6 and 12 months follow-ups;
- Function at all time points, measured with the Patient Specific Functional Scale [30], a self-reported scale specific for the measurement of functionality, where the patient nominates activities relevant to them and rates their ability to perform each activity on a 0 to 10 scale, with 0 representing “unable to perform activity” and 10 represents “able to perform at the same level as before injury or problem”;
- Health-related quality of life at all time points, measured with the SF-12 [31], a self-reported questionnaire with 12 questions and classification of 8 different dimensions related to quality of life. Higher scores reflect better quality of life;
- Kinesiophobia at all time points, measured with the Tampa Scale of Kinesiophobia [32], a self-reported questionnaire with 17 items scored on a four point likert agreement scale (“totally disagree” “partially disagree”, “partially agree”, “totally agree”. Three items [4, 8, 12] are reverse scored. Higher scores on Tampa reflect higher kinesiophobia;
- Global perceived effect at all time points, measured with the Global Perceived Effect Scale [30], a self-reported scale, with scores of − 5 to + 5, where the progression of the patient’s condition will be classified from a certain point in time. Scores of − 5 indicate the condition is vastly worse while scores of + 5 indicates completely recovered.
- Anxiety and depression at all time points, measured by the Hospital Anxiety and Depression Scale [33], a self-reported scale with 14 items, 7 for depression and 7 for anxiety. Each item is scored on 0 to 3 scale. Higher scores indicate higher depression or anxiety.
- Pain catastrophisation at all time points, measured with the Pain Catastrophising Scale [34], a self-reported scale with 13 statements, where the patient ranks the affirmations between 0 being “minimal” to 4 “very intense.” The calculation of the final score is based on 3 subdomains of the scale and higher scores represent higher catastrophising.
- Pain-related attitudes and beliefs at all time points, measured with the Short Form Orebro Musculoskeletal Pain Questionnaire [35], with 10 items related to musculoskeletal pain each scored from 0 to 10 with total score ranging from 0 to 100. Patients with scores between 51 and 100 points are classified as high risk of developing long-term disability.
- Self-efficacy at all time points, measured with Pain Self-Efficacy Questionnaire [36], a questionnaire with 22 questions classified in three domains, with a score of each domain ranging from 10 to 100. A total score close to 300 indicates a greater sense of self-efficacy.
- Adverse events measured by recording the number of adverse events during the intervention period.

We will also assess the patient’s expectancy for improvement at baseline using the Expectancy of Improvement Numerical Scale [37].

The same outcome measures will be used at the baseline assessment, post-intervention (8 weeks), 6 and 12 months after randomisation.

Eligible participants will be randomly allocated to one of the treatment groups through an electronic platform. After baseline screening, patients will receive a login and password to access the study website. When the patient logs on to the website, he/she will be randomly allocated to one of the two study groups. The assessor who will confirm the eligibility will be unaware about patients’ allocation.

### Blinding

The outcome assessor will be blinded to the treatment groups. The assessor will be asked to guess which group the patients were in (intervention or control) at the end of the study to measure assessor blinding. Due to the nature of the interventions, it will be not possible to blind neither the patients nor treatment providers.

### Interventions

#### Internet-based pain education and exercise

Patients allocated to the intervention group will receive a login and password for individual access to the website designed for the study (www.reabilitador.com.br). The content of this intervention will include videos and animations based on pain education, physical activity promotion and general exercises. The pain education component will be based on the E-pain intervention developed by Reis at al [38], .which includes nine main features: (1) acceptance, (2 and 3) pain education, (4) sleep hygiene, (5) recognising stress and negative emotions, (6) increasing positive coping in lifestyle, (7) exercises, (8) communication and (9) prevention. The exercise program was created by professional physiotherapists with at least 5 years of clinical experience, specialists in the treatment of chronic pain and who used exercise-based treatment. After its elaboration, it was submitted to a round of suggestions and adjustments to the program by a panel of experts. After this round of suggestions, the exercise component was sent by email to a group of experts in the chronic pain field. After the last round of suggestions and corrections, the exercise program was modified to be simple and assertive for the Brazilian population. The exercise component includes general exercises aiming to improve strength, flexibility, control and coordination.

The total duration of the intervention will be 8 weeks. There will be new content each week of the intervention and patients will be instructed to perform the exercises from the video at least three times per week and watch the videos as much as needed. Patients in this group will also receive weekly text messages and health coaching over the telephone. The text messages will include information on the benefits of exercises, motivation, and positive messages about dealing with pain. The health coaching will be performed once a week until the end of the intervention (8 weeks) by a physiotherapist (5 years of experience) with previous training for the coaching. The aim of the health coaching component is to keep patients motivated to engage with the program. This will include encouragement, motivation, coping, review of the instructions, and if necessary, tailoring of the content of the intervention. For example, if a patient feels any discomfort while doing an exercise, the coach will slightly modify the exercise (e.g. dose, range of motion). Adherence to the program will be recorded in a daily log.

##### Control group (online booklet)

The patients allocated to the control group will have access to an online booklet containing general information about self-management of chronic pain, including pain education, advice on healthy lifestyle and sleeping habits and promotion of physical activity. They will also receive one phone call at week 4 and motivational text messages once a week during the study period.

### Economic evaluation

The economic evaluation will be conducted from a societal perspective over 12 months (at the 8 week, 6, 9 and 12 months follow-ups). Intervention costs will be determined by the real costs of maintenance and support, monitoring costs, training and project management costs, number and length of telephone calls, and number of text messages sent to participants. Costs associated with preparing the organisation and developing the website and video recordings will be excluded. We will measure costs by the estimate of healthcare utilisation costs (public and private if possible), patients costs, and lost productivity costs (absenteeism) collected using a questionnaire. Costs will be measured based on the participants’ reported use of the resources using a cost diary given to the participants at baseline.

The healthcare utilisation costs incurred due to chronic pain (e.g., medication, visits to health professionals, hospital stay, visits to emergency departments, diagnostic tests) will be valued using the Brazilian standard costs [39]. The patient costs include costs of community services (e.g., gym), out-of-pocket expenses, over-counter medication, and transportation (private car and/or public transport) and will be based on the self-reported costs of participants. Transportation by car will be valued using Brazilian gasoline prices, and public transportation using the reference price of the city of São Paulo. The lost productivity costs will include absenteeism from work (paid and unpaid) and costs from transportation (private car and /or public transport). The costs of absenteeism from work (paid and unpaid work) and will be valued using gender-specific price weights [40]. Transportation by car will be valued using Brazilian gasoline prices, and public transportation using the reference price of the city of São Paulo.

We will perform cost-effectiveness and cost-utility analyses according to the intention-to-treat principles [41]. Differences in costs and effects will be estimated using seemingly unrelated regression analyses. The incremental cost-effectiveness ratios will be calculated by the difference between intervention costs divided by the difference between intervention effects. Bootstrapping techniques will be performed to estimate uncertainty surrounding the cost difference and incremental cost-effectiveness ratios. The cost-effectiveness analysis will be conduct for pain intensity at 12 months after randomisation. The cost-utility analysis will be conducted using the health utility index measured by the SF-6D (derived from the SF-12) to calculate the quality-adjusted life-years (QALY). Two exploratory sensitivity analysis will be performed, one including the total costs of the intervention (including costs with preparing and developing the website and videos) diluted in a 10-year period, considered as the available period of use of the resource. The second sensitivity analysis will be performed considering only patients with more than 75% of adherence to the program.

### Statistical methods

#### Sample size calculation

We determined that a minimum of 160 individuals (80 per group) would be required to provide the trial with 90% power to detect a between-group difference of 1.5 point in a 0 to 10 Pain Numerical Rating Scale, with an estimated standard deviation of 2.75 points and two-sided alpha level of 0.05. The estimated sample size would also allow for loss to follow up rate of up to 15%.

#### Data analysis

The normality of the data will be tested by visual inspection of histograms. Baseline characteristics of the participants will be calculated using descriptive statistics. The between-group differences and 95% confidence interval for the post-treatment outcomes at 8 weeks, 6- and 12-months follow-ups will be calculated using Mixed Linear Models using interaction terms of treatment group versus time. An intention-to-treat approach will be used in all statistical analyses.

### Mediation analysis

We will use causal mediation analysis to understand the mechanisms by which the internet-based pain education and exercise exerts its effects on the primary outcome, pain intensity at 8 weeks. The proposed intervention aims to reduce kinesiophobia, pain catastrophising, and improve self-efficacy, and pain-related attitudes and beliefs post-treatment. It is hypothesised that short term effects of these putative mediators will cause reduction in pain intensity at 8 weeks. We will estimate indirect effects of the intervention via the putative mediators, and path specific effects (intervention-mediator and mediator-outcome). We will use directed acyclic graphs to identify the minimum sufficient set of confounders that will need to be adjusted. These directed acyclic graphs will be registered before data collection begins. We will use sensitivity analyses to explore how much residual confounding would explain away the indirect effect, if one is detected. We will use the ‘mediation’ R package to conduct these analyses [42].

## Discussion

We hypothesise that the telerehabilitation program will benefit patients with chronic musculoskeletal pain by reducing pain intensity and improving function, and psychological and behavioral outcomes (i.e., anxiety, depression, fear-avoidance, catastrophising) compared an online minimal intervention approach (booklet). We also hypothesise that the telerehabilitation program will be a cost-effective intervention compared to the control intervention for patients with chronic musculoskeletal pain. As patients usually support the use of telerehabilitation to improve access to care, we expect that the program will be well received by the patients.

The program is based on relatively simple interventions (i.e., home-based exercise and pain education), although some support is necessary from the coach. This program was designed to be simple to facilitate its implementation in healthcare settings. The program has the potential to change lives of millions of people with chronic musculoskeletal pain that are everyday waiting for treatment in overpopulated cities in Brazil. If the results of our study prove our hypothesis about the effects of treatment on patients with chronic pain, we can contribute to the discussion of a new perspective of this treatment modality for public and supplementary health. The results of the study may serve as a basis for decision making and possible implementation of this therapeutic modality in the treatment of chronic musculoskeletal pain. The economic evaliation present in the study may provide an observation of an important aspect for the implementation of telerehabilitation in the health system. The possibility of reducing health costs would also make it possible to reallocate investments in healthcare.

## Supplementary information

**Additional file 1.**

## Data Availability

All data will be used only for analysis of the present study and will be protected from any unnecessary exposure. The Consent Form according to the Research Ethics Committee of Universidade Cidade de São Paulo was signed by the authors and patients. The paper information will be kept in binders that will be handled only by the researchers responsible for the study and will be kept in locations that will be accessible only to researchers responsible for data analysis. The online information will be able to the researchers in a personal computer located in the personal room of the researcher responsible for the project. All online information will be assessed just by the responsible researchers of specific part of the data analysis. All information will be published confidentially, without the name of the subjects exposed. All data will be available for review and confirmation of data analysis when requested by a review process for publication of the article in indexed scientific journals or presentations at scientific events.

## Abbreviations

SPIRIT: Standard Protocol Items: Recommendations for Interventional Trials.
CONSORT: Consolidated Standards of Reporting Trials
