# Supplementary material for: Evaluation of the efficacy of an internet-based pain education and exercise program for chronic musculoskeletal pain in comparison with online self-management booklet: a protocol of a randomised controlled trial with assessor-blinded, 12-month follow-up, and economic evaluation
Source: BMC Musculoskelet Disord. 2020 Jun 26;21:404. doi: 10.1186/s12891-020-03423-x (PMC7320555; doi:10.1186/s12891-020-03423-x)
Supplement: Supplementary file 1 — Additional file 1. [file 12891_2020_3423_MOESM1_ESM.zip › Human Research Ethics Committee ApprovalR1.docx]

**Human Research Ethics Committee**

**Title**: Evaluation of the Efficacy of an Internet-Based Pain Education and Exercise Programme for Chronic Musculoskeletal Pain in Comparison with Online Self-Management Booklet: a Randomised Controlled Trial with Assessor-Blinded, 12-month Follow-up, and Economic Evaluation

**Principal Researcher**: Iuri Fioratti / **Supervisor**: Bruno Tirotti Saragiotto

**Project ID (CAAE)**: 02892918.0.0000.8084

**Institution**: Universidade Cidade de São Paulo

**Situation**: Approved (20 December 2018)

**Documents Approved**:

| **Type** | **Document** |
| --- | --- |
| Participant Consent Form | Consent Form |
| Project | Research project |
| Basic Information on the Project | Folha de Rosto |
| Timeline | Project timeline |

**Chief Investigator / Supervisor’s responsibilities:**

1. You must retain copies of all signed Consent Forms (if applicable) and provide these to the Human Research Ethics Committee on request.

2. It is your responsibility to provide a copy of this letter to any internal/external granting agencies if requested.

Yours sincerely,


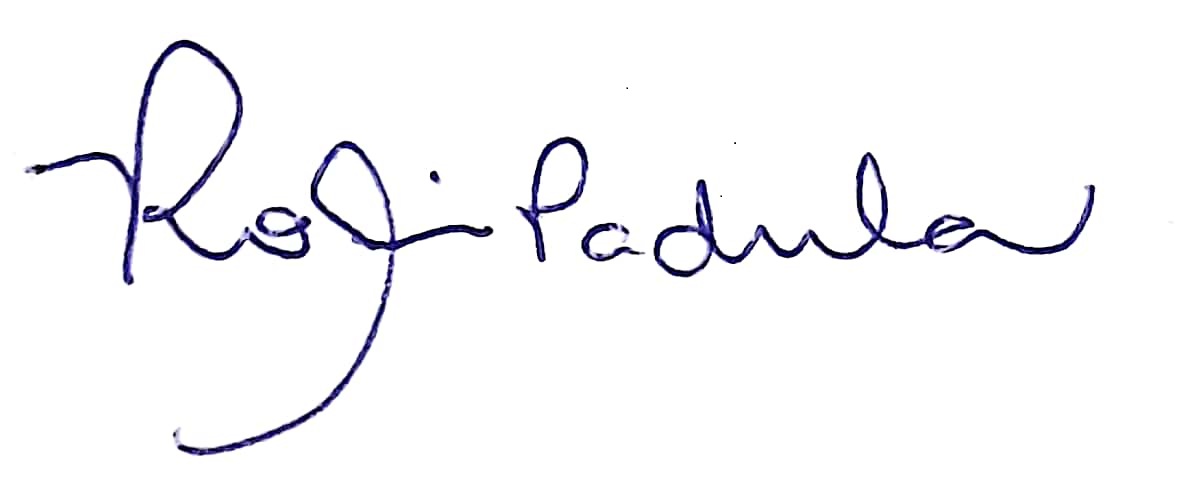


________________________________________

Rosimeire Simprini Padula

Head of Department, Masters and Doctoral Program in Physical Therapy

Universidade Cidade de São Paulo
