# Supplementary material for: Evaluation of the efficacy of an internet-based pain education and exercise program for chronic musculoskeletal pain in comparison with online self-management booklet: a protocol of a randomised controlled trial with assessor-blinded, 12-month follow-up, and economic evaluation
Source: BMC Musculoskelet Disord. 2020 Jun 26;21:404. doi: 10.1186/s12891-020-03423-x (PMC7320555; doi:10.1186/s12891-020-03423-x)
Supplement: Supplementary file 1 — Additional file 1. [file 12891_2020_3423_MOESM1_ESM.zip › PB_PARECER_CONSUBSTANCIADO_CEPR1_3098415.pdf]

PARECER CONSUBSTANCIADO DO CEP

DADOS DO PROJETO DE PESQUISA

Título da Pesquisa: Efetividade e Custo-Efetividade de um Programa de Tele-Reabilitação no Tratamento da Dor Crônica Musculoesquelética

Pesquisador: IURI FIORATTI

Área Temática: Equipamentos e dispositivos terapêuticos, novos ou não registrados no País;

Versão: 2

CAAE: 02892918.0.0000.8084

Instituição Proponente: SECID - SOCIEDADE EDUCACIONAL CIDADE DE SAO PAULO LTDA

Patrocinador Principal: UNIVERSIDADE DE SAO PAULO

DADOS DO PARECER

Número do Parecer: 3.098.415

Apresentação do Projeto:

Trata de um projeto de doutorado do aluno Iuri Fioratti, sob a orientação da Prof. Dr. Leonardo Oliveira Pena Costa do Programa de Pós Graduação em Fisioterapia da Universidade de São Paulo. O presente estudo pretende investigar a efetividade e custo efetividade de um programa de tele-reabilitação para pacientes com dor crônica musculoesquelética. A proposta inicial foi submetida 01/11/2018, retornando com as seguintes pendencias: 1) Informar as formas de minimização dos riscos nas IBPs e TCLE; 2) Corrigir a gramatica, formatação do TCLE e acrescentar o contato telefônico dos pesquisadores e do CEP; 3) Acrescentar a capa no projeto e 4) Ajustar e sincronizar o cronograma em todos os documentos apresentados. Nessa submissão (05/12/2018), todas as pendencias foram adequadamente respondidas e justificadas. O inicio da coleta de dados esta previsto para 01/02/2019.

Objetivo da Pesquisa:

O objetivo do estudo será desenvolver e testar a eficácia de um programa de autogerenciamento baseado em exercícios e educação em dor pela Internet comparado com uma intervenção mínima para pessoas com dor crônica musculoesquelética em São Paulo, Brasil. O seu objetivo secundário será observar se há relação positiva de custo-efetividade e custo-utilidade do programa de autogerenciamento pela internet comparado com a intervenção mínima.

Avaliação dos Riscos e Benefícios:

Foi informado que o programa/pesquisa é baseado em intervenções relativamente simples (ou seja, exercícios domiciliares e educação), embora seja necessário algum apoio do terapeuta. Este programa foi projetado para ser simples para facilitar sua implementação em ambientes de saúde pública. Apesar de acreditarmos que o risco de prejuízos ou danos à saúde decorrente da participação neste estudo é extremamente baixo, acreditamos também que não há estudo envolvendo humanos que seja 100% livre de riscos. Todas as intervenções que serão realizadas no estudo já foram testadas pela comunidade científica e apresentam riscos baixos de intercorrências e complicações. Por se tratar de um programa de exercícios físicos, é possível que ocorram sintomas comuns da prática de exercícios, como dor muscular, fadiga ou cansaço. Os pesquisadores do estudo serão comunicados sobre quaisquer intercorrências que possam acontecer. Para minimizar estes riscos, o estudo contará com um acompanhamento semanal por meio de ligações telefônicas que poderão ser utilizadas para esclarecer quaisquer dúvidas em relação aos exercícios realizados e aconselhamento sobre estes possíveis sintomas adversos e a melhor conduta a ser tomada.

Acredita-se que o conteúdo dos dois grupos de tratamento tem potencial de ajudar o paciente a gerenciar a sua condição de dor crônica. Além disso, este estudo busca entender mais sobre os tratamentos para dor crônica e tem potencial de beneficiar milhares de pessoas com esta condição no mundo. Os resultados deste estudo podem ajudar a melhorar o manejo da dor crônica no sistema público de saúde, reduzindo custos e permitindo que os recursos sejam direcionados em condições de saúde onde não é possível implementar esse tipo de abordagem (por exemplo, emergência, quimioterapia, infecção, doença cardíaca e condições neurológicas agudas.

Comentários e Considerações sobre a Pesquisa:

No Brasil, dor crônica tem sido reportada como o principal motivo em que pacientes procuram serviços de pronto atendimento em hospitais, o que muitas vezes sobrecarrega o sistema de saúde. Uma intervenção promissora que tem sido utilizada em estudos prévios é a tele-reabilitação. Tele-reabilitação é realizada através do uso de tecnologias (internet ou outros meios digitais) para oferecer tratamento. Isto pode facilitar o acesso ao tratamento para estes pacientes, superando barreiras como transporte, filas de espera, falta de seguro saúde e altos custos para tratamentos. Portanto, o objetivo principal deste projeto de pesquisa é de investigar a efetividade e custo-efetividade de um programa de tele-reabilitação para pacientes com dor crônica musculoesquelética. Os 160 pacientes serão divididos aleatoriamente entre um grupo de tratamento por tele-reabilitação (exercício e educação em dor) ou grupo controle (intervenção

mínima). O desfecho primário será intensidade da dor, e os desfechos secundários incluirão função e desfechos psicossociais. Todos os desfechos serão avaliados no final do tratamento (8 semanas) e após 3, 6 e 12 meses após a aleatorização. Será utilizado um Modelo Linear Misto para a análise estatística. O programa de tele- reabilitação proposto neste projeto tem o potencial de beneficiar pacientes com dor musculoesquelética que geralmente esperam por tratamento nos sistemas de saúde através de uma intervenção simples e de fácil acesso.

Considerações sobre os Termos de apresentação obrigatória:

Nas IBPs:

- Informa os riscos e benefícios do programa de tratamento de tele-reabilitação, assim como apresenta meios de minimização dos riscos.
- Apresenta os procedimentos de pesquisa de forma clara;
- Apresenta o cronograma compatível com a proposta do estudo, com inicio previsto para 01/02/2018.

No TCLE

- O TCLE está escrito e direcionado aos participantes, descreve os procedimentos de forma clara e acessível;
- Apresenta o título e objetivo do estudo;
- Apresentada informação sobre contato do orientador e aluno/pesquisador, com contato telefônico;
- Apresenta de forma apropriada os riscos da participação no estudo e informa as formas de minimização dos mesmos;
- Apresenta de forma apropriada os benefícios do estudo;
- Finaliza o TCLE na forma de convite e solicita, caso de acordo, a assinatura do mesmo por parte do responsável.

Outros documentos apresentados:

- Formulário de encaminhamento de pesquisa;
- A folha de rosto está devidamente preenchida, assinada e carimbada;
- Cronograma.

Recomendações:

Não há.

Conclusões ou Pendências e Lista de Inadequações:

A presente proposta foi analisada e está devidamente apresentada e justificada. Todas as pendências inicialmente levantadas foram devidamente respondidas. Assim, o presente parecer é pela aprovação da mesma.

Considerações Finais a critério do CEP:

O Colegiado do CEP acompanha o parecer do relator.

O presente projeto, seguiu nesta data para análise da CONEP e só tem o seu início autorizado após a aprovação pela mesma.

Este parecer foi elaborado baseado nos documentos abaixo relacionados:

Tipo Documento	Arquivo	Postagem	Autor	Situação
Informações Básicas do Projeto	PB_INFORMAÇÕES_BÁSICAS_DO_PROJETO_1218769.pdf	05/12/2018 11:52:06		Aceito
TCLE / Termos de Assentimento / Justificativa de Ausência	tcle_doutorado_cep.pdf	05/12/2018 11:49:33	Bruno Tirotti Saragiotto	Aceito
Projeto Detalhado / Brochura Investigador	projeto_de_pesquisa.docx	05/12/2018 11:48:30	Bruno Tirotti Saragiotto	Aceito
Cronograma	cronograma_projeto.docx	05/12/2018 11:26:49	IURI FIORATTI	Aceito
Outros	formulario_cep.pdf	30/10/2018 19:55:22	IURI FIORATTI	Aceito
Folha de Rosto	folha_de_rosto.pdf	13/09/2018 19:32:46	IURI FIORATTI	Aceito

Situação do Parecer:

Aprovado

Necessita Apreciação da CONEP:

Sim

SAO PAULO, 20 de Dezembro de 2018

Assinado por:

Sandro Massao Hirabara
(Coordenador(a))

Endereço: Av. Ussiel Cirilo, nº 225, São Paulo / SP
Bairro: Vila Jacui **CEP:** 08.060-070
UF: SP **Município:** SAO PAULO
Telefone: (11)2037-5805 **Fax:** (11)2037-5805 **E-mail:** cep@cruzeirodosul.edu.br
