# Supplementary material for: Evaluation of the efficacy of an internet-based pain education and exercise program for chronic musculoskeletal pain in comparison with online self-management booklet: a protocol of a randomised controlled trial with assessor-blinded, 12-month follow-up, and economic evaluation
Source: BMC Musculoskelet Disord. 2020 Jun 26;21:404. doi: 10.1186/s12891-020-03423-x (PMC7320555; doi:10.1186/s12891-020-03423-x)
Supplement: Supplementary file 1 — Additional file 1. [file 12891_2020_3423_MOESM1_ESM.zip › termo_de_outorga_-_processo-2016_24217-7R1.pdf]

FAPESP

Para uso exclusivo da FAPESP
Processo: 2016/24217-7
Data impressão: 12/04/2018 12:23:46
Folha: ________________
Volume: _______________
Rubrica: _______________

FUNDAÇÃO DE AMPARO À PESQUISA DO ESTADO DE SÃO PAULO

TERMO DE OUTORGA E ACEITAÇÃO DE AUXÍLIOS

PROCESSO 2016/24217-7

Pelo presente instrumento, a Fundação de Amparo à Pesquisa do Estado de São Paulo, com sede na Rua Pio XI, n° 1.500, Alto da Lapa, São Paulo, Capital, inscrita no CNPJ/MF sob nº 43.828.151/0001-45, doravante denominada OUTORGANTE, por meio de seu Conselho Técnico-Administrativo, nos termos do Artigo 14, letra "b", da Lei Estadual nº 5.918, de 18 de outubro de 1960, concede ao OUTORGADO, a seguir qualificado, Auxílio para a realização do Projeto de Pesquisa a seguir especificado, nas instalações e com o apoio da INSTITUIÇÃO SEDE, de acordo com as especificações, cláusulas e condições descritas a seguir e nos Anexos, que passam a ser parte integrante deste Termo.

**1.OUTORGADO:**	Bruno Tirotti Saragiotto<br>CPF: 368.641.358-29<br>RG: 43522315-SP/SSP/SP
**2.Correspondência:**	Rua Sacadura Cabral, 160, apto 51 Jatobá - Apto 51 Jatobá, Lapa, São Paulo/SP, CEP 05076-020<br>bruno.saragiotto@gmail.com
**3.Instituição Sede:**	Pró-Reitoria de Pós-Graduação, Pesquisa e Extensão/PRPGPE<br>Universidade Cidade de São Paulo/UNICID
**4.Projeto de Pesquisa:**	A efetividade de um programa de tele-reabilitação para o tratamento da dor crônica musculoesquelética: Programa e-Rehab
**5.Linha de Fomento:**	Programas Especiais / JP - Programa Jovens Pesquisadores em Centros Emergentes / Auxílio à Pesquisa - Jovem Pesquisador
**6.Área/Subárea:**	Fisioterapia e Terapia Ocupacional<br>Outra Subárea Fisioterapia e Terapia Ocupacional
**7.Coordenação:**	Saúde III
**8.Período da Vigência:**	01/04/2018 a 31/03/2022
**9.Relatórios Científicos:**	20/09/2019, 20/01/2021, 20/04/2022
**10.Prestações de Contas:**	20/03/2019, 20/03/2020, 20/03/2021, 20/04/2022

CLÁUSULA PRIMEIRA - DA NATUREZA DO BENEFÍCIO:

1.1. O presente Termo de Outorga não corresponde a qualquer espécie de relação de emprego entre o OUTORGADO e a OUTORGANTE, uma vez que não configura vínculo trabalhista, nem objetiva pagamento de salário, não se estendendo ao OUTORGADO benefícios exclusivos dos servidores da OUTORGANTE.

1.2. A OUTORGANTE não se responsabilizará por cobrir despesas de assistência médica e odontológica de qualquer natureza, excetuando-se seguro-saúde, quando concedido.

CLÁUSULA SEGUNDA - DOS RECURSOS FINANCEIROS DISPONIBILIZADOS:

2.1. A utilização dos recursos deverá obedecer às normas constantes do Anexo III deste Termo de Outorga, bem como às regras relativas à utilização da Reserva Técnica, constante do Anexo V deste Termo de Outorga.

2.2. É vedado ao OUTORGADO contratar ou destinar verbas concedidas para a execução do projeto, a que título for, a pessoas:

2.2.1. Físicas que sejam parentes (ascendentes, descendentes ou colaterais até o 4º grau, consanguíneos ou por afinidade), cônjuges ou companheiros do OUTORGADO ou de membro de sua equipe de pesquisa bem como servidores, colaboradores da FAPESP, ainda que eventuais, e membros do Conselho Superior.

2.2.2. Jurídicas que tenham como sócios o próprio OUTORGADO, membros de sua equipe de pesquisa, servidores ou colaboradores da FAPESP, ainda que eventuais, e membros do Conselho Superior, bem como parentes (ascendentes, descendentes ou colaterais até o 4º grau, consanguíneo ou por afinidade), o cônjuge ou companheiro de quaisquer deles.

2.3. As liberações dos recursos serão feitas em conta específica vinculada à OUTORGANTE.

2.3.1. O OUTORGADO deverá solicitar a liberação dos recursos somente quando houver necessidade imediata da realização dos gastos, evitando assim a permanência de saldo na conta, não sendo permitida aplicação no mercado financeiro.

2.4. O Auxílio concedido não poderá ser destinado, em hipótese alguma, ainda que parcialmente, a fins diversos dos indicados no preâmbulo deste Termo de Outorga, ficando o OUTORGADO pessoalmente responsável pela sua perfeita utilização, em conformidade com os dispositivos legais vigentes.

2.5. Para todas as utilizações de recursos previstos, inclusive para fins de pagamento de manutenção ou diárias, a OUTORGANTE considera o período previsto neste Termo de Outorga, exceto no caso dos Equipamentos Multiusuários.

2.5.1. Em Auxílios à Pesquisa - Organização de Reunião Científica e/ou Tecnológica e Auxílios à Pesquisa - Participação em Reunião Científica e/ou Tecnológica a liberação de verba poderá ser solicitada até 30 dias antes do início da vigência.

2.5.2. A utilização de recursos para aquisição de Equipamento Multiusuário (EMU) deverá ser feita no prazo máximo de até 3 (três) anos, contados da data de início do projeto determinada no Termo de Outorga e Aceitação de Auxílios. Após decorrido esse prazo a verba para tanto destinada não será mais disponibilizada.

2.5.3. Nas hipóteses de aquisição de Equipamentos Multiusuários deverá ser entregue anualmente um Relatório Científico até o 7º ano após a data de início da vigência, em que se destaque a utilização do EMU no desenvolvimento da pesquisa.

2.5.4. Caso haja despesas efetuadas fora do período de vigência, fica o OUTORGADO obrigado a efetuar a devolução à OUTORGANTE do valor despendido fora das condições estabelecidas.

CLÁUSULA TERCEIRA - DA CONTRAPARTIDA EXTERNA EM PROJETOS DE PESQUISA:

3.1. Se o projeto envolver desembolso direto de recursos de terceiros para a INSTITUIÇÃO SEDE, deverá ser celebrado um Termo de Convênio juntamente com este Termo de Outorga.

3.1.1. O Termo de Convênio deverá ser assinado pela INSTITUIÇÃO SEDE, a entidade parceira e a OUTORGANTE, com a ciência do OUTORGADO.

3.2. No Termo de Convênio deverá ser explicitado o valor total do Convênio, o cronograma e a forma dos desembolsos da entidade parceira e, se for o caso, da Instituição Sede, além dos prazos e instruções quanto à comprovação de tais desembolsos, bem como as cláusulas sobre o tratamento da Propriedade Intelectual.

3.2.1. O acordado entre as partícipes no Termo de Convênio poderá afetar as Cláusulas Segunda, Sexta e Oitava deste Termo de Outorga.

CLÁUSULA QUARTA - DA PARTICIPAÇÃO, DO REGIME DE DEDICAÇÃO E DAS OUTRAS OBRIGAÇÕES DO OUTORGADO:

4.1. O OUTORGADO se obriga a:

4.1.1. Dedicar-se integralmente à execução do projeto pelo tempo declarado na proposta analisada e aprovada pela OUTORGANTE.

4.1.2. Consultar a OUTORGANTE antes de aceitar qualquer apoio financeiro de qualquer outra fonte de financiamento, pública ou privada, para o desenvolvimento do mesmo projeto de pesquisa a que concerne o Auxílio concedido.

4.1.3. Consultar a OUTORGANTE antes de fazer quaisquer modificações no projeto, incluindo, mas não restritas a, aquelas no plano inicial, nas datas ou na designação de recursos.

4.1.4. Consultar a OUTORGANTE antes de assumir compromisso que exija seu afastamento da INSTITUIÇÃO SEDE por mais de 90 dias.

4.1.5. Apresentar os Relatórios Científicos e as Prestações de Contas de progresso e final, nos prazos estipulados neste Termo de Outorga e em conformidade com as normas institucionais da OUTORGANTE, sob pena de ser acionado administrativa e/ou judicialmente pela OUTORGANTE para devolução dos recursos recebidos, devidamente corrigidos pelos índices legais em vigor e com incidência das demais sanções legais (juros, honorários advocatícios e custas judiciais).

CLÁUSULA QUINTA - DO TRATAMENTO E GUARDA DO MATERIAL PERMANENTE E DE CONSUMO:

5.1. A aquisição de material de consumo ou permanente nacional e/ou importado deverá obedecer às normas constantes do Anexo III deste Termo de Outorga, sob pena de ser acionado administrativa e/ou judicialmente pela OUTORGANTE para devolução dos recursos recebidos, devidamente corrigidos pelos índices legais em vigor e com incidência das demais sanções legais (juros, honorários advocatícios e custas judiciais).

5.2. Em caso de sinistro (roubo, furto, avaria ou outro) envolvendo equipamentos destinados à execução do

projeto de pesquisa e adquiridos com recursos da OUTORGANTE, o OUTORGADO deverá lavrar boletim de ocorrência e comunicar imediatamente o fato à OUTORGANTE.

5.2.1. Quando o evento/sinistro ocorrer nas dependências da INSTITUIÇÃO SEDE, o OUTORGADO compromete-se a solicitar a instauração de sindicância administrativa para apuração da autoria do fato, remetendo à OUTORGANTE cópia da portaria instauradora da sindicância e, posteriormente, cópia do Relatório Final da Sindicância.

CLÁUSULA SEXTA - DAS PRESTAÇÕES DE CONTAS E DOS RELATÓRIOS CIENTÍFICOS:

6.1. As Prestações de Contas do Auxílio concedido serão feitas pelo OUTORGADO em conformidade com as instruções dos Anexos III e V deste Termo de Outorga, na(s) data(s) de vencimento indicada(s) no Campo 10 do preâmbulo, devendo, nesse momento, efetuar a devolução, em cheque ou depósito bancário identificado, do saldo , se houver.

6.1.1. As Prestações de Contas serão recebidas pela OUTORGANTE, ficando sua aprovação condicionada à emissão de parecer favorável pela FAPESP.

6.2. Independentemente das Cláusulas 6.1 e 6.1.1, e em conformidade com as normas da respectiva modalidade de Auxílio, o OUTORGADO se obriga a apresentar à OUTORGANTE, nas datas indicadas no Campo 9 do preâmbulo, os Relatórios Científicos da pesquisa com conclusões sucintas dos resultados até então obtidos.

6.2.1. A continuidade do apoio da OUTORGANTE ao projeto ou seu efetivo encerramento estão condicionados à aprovação dos Relatórios Científicos de Progresso e Relatório Científico Final.

CLÁUSULA SÉTIMA - DAS PUBLICAÇÕES E DIVULGAÇÕES:

7.1. O OUTORGADO se compromete a fazer referência ao apoio da OUTORGANTE em todas as formas de divulgação (teses, dissertações, artigos, livros, resumos de trabalhos apresentados em reuniões, páginas na Web e qualquer outra publicação ou forma de difusão de atividades) que resultem, total ou parcialmente, de Auxílio ou Bolsa objeto deste Termo de Outorga.

7.1.1. O OUTORGADO deverá indicar, em cada publicação prevista na cláusula 7.1, além do nome FAPESP, o número do processo FAPESP a que se refere este Termo de Outorga, no modelo: processo nº aaaa/nnnnn-d, Fundação de Amparo à Pesquisa do Estado de São Paulo (FAPESP).

7.1.1.1. Os artigos escritos em idioma estrangeiro deverão indicar o apoio da FAPESP em inglês, conforme o seguinte modelo: grant #aaaa/nnnnn-d, São Paulo Research Foundation (FAPESP).

7.2. O OUTORGADO é responsável por garantir que em toda publicitação de materiais (incluindo páginas web) que resultem total ou parcialmente de Auxílio ou Bolsa objeto deste Termo de Outorga, exceto artigos científicos publicados em revistas científicas ou técnicas com revisão por pares, conste a seguinte declaração de responsabilidade: "As opiniões, hipóteses e conclusões ou recomendações expressas neste material são de responsabilidade do(s) autor(es) e não necessariamente refletem a visão da FAPESP".

CLÁUSULA OITAVA - DO TRATAMENTO DA PROPRIEDADE INTELECTUAL:

8.1. O OUTORGADO compromete-se a verificar, em tempo hábil, se a execução do projeto produz ou poderá

produzir resultado potencialmente, no todo ou em parte, objeto de proteção por Patente de Invenção, Modelo de Utilidade, Desenho Industrial, Software ou qualquer outra forma de proteção dos direitos de Propriedade Intelectual, observadas as normas constantes do Anexo IV deste Termo de Outorga.

8.2. O OUTORGADO e a INSTITUIÇÃO SEDE declaram estar cientes de que a titularidade ou cotitularidade dos direitos de Propriedade Intelectual fica estabelecida conforme os seguintes critérios especificados nos itens 3.1, 3.2, 3.3 e 3.4 do Anexo IV deste Termo de Outorga:

8.2.1 A INSTITUIÇÃO SEDE, quando esta possuir, no momento do requerimento da proteção, órgão formalmente constituído para gerir sua política para Propriedade Intelectual e inovação tecnológica (doravante NIT), devidamente qualificado pela OUTORGANTE; ou

8.2.2. A OUTORGANTE e a INSTITUIÇÃO SEDE, quando a INSTITUIÇÃO SEDE não tiver NIT qualificado no momento do requerimento da proteção;

8.2.3 No âmbito do Programa PIPE, a INSTITUIÇÃO SEDE, no caso do OUTORGADO possuir vínculo empregatício com a empresa, ou a OUTORGANTE, no caso do OUTORGADO ser bolsista da FAPESP;

8.2.4 A OUTORGANTE e/ou a INSTITUIÇÃO SEDE e terceiros, de acordo com o previsto em contrato ou convênio firmado entre as partes ou apresentado como condição para liberação do benefício.

8.3. O OUTORGADO e a INSTITUIÇÃO SEDE declaram estar cientes de que a OUTORGANTE, independentemente do custeio do registro, terá participação sobre os benefícios decorrentes da exploração dos direitos de Propriedade Intelectual, nos termos da Política para Propriedade Intelectual da FAPESP, Anexo IV deste Termo de Outorga.

8.3.1. Caberá à INSTITUIÇÃO SEDE, de acordo com suas políticas e a legislação aplicável, definir internamente a distribuição dos benefícios entre o OUTORGADO e os demais envolvidos na obtenção do resultado.

CLÁUSULA NONA - DO COMPROMISSO DE EMISSÃO DE PARECER DE ASSESSORIA EM TEMPO HÁBIL:

9.1. Em decorrência do Auxílio que lhe foi concedido, o OUTORGADO se compromete a emitir pareceres técnicos e científicos em assuntos de sua especialidade, quando solicitados pela OUTORGANTE, gratuitamente e dentro do prazo estipulado pela OUTORGANTE.

9.1.1. A não observância do disposto na cláusula 9.1 poderá acarretar bloqueio parcial ou total de recursos de Auxílios e Bolsas sob a responsabilidade do OUTORGADO em andamento na OUTORGANTE.

CLÁUSULA DÉCIMA - DO COMPROMISSO DE OBSERVÂNCIA DA LEGISLAÇÃO RELATIVA À PROTEÇÃO DA VIDA HUMANA, UTILIZAÇÃO DE ANIMAIS, MANIPULAÇÃO DE ORGANISMOS GENETICAMENTE MODIFICADOS, UTILIZAÇÃO DE MATERIAL NUCLEAR, BIODIVERSIDADE, PESQUISA EM TERRAS INDÍGENAS E CONHECIMENTOS TRADICIONAIS ASSOCIADOS BEM COMO DAS DEMAIS EXIGÊNCIAS LEGAIS APLICÁVEIS:

10.1. Declaram o OUTORGADO e a INSTITUIÇÃO SEDE que é de sua exclusiva responsabilidade solicitar, obter, possuir e demonstrar, quando solicitado pela OUTORGANTE, todas as autorizações legais e exigíveis para a boa execução do projeto, que deverão ser emitidas pelos Órgãos de controle e fiscalização atinentes à natureza da pesquisa quando assim for exigido.

10.2. As declarações a que se refere o item anterior incluem, mas podem não se limitar, àquelas emitidas por: Instituto Brasileiro de Meio Ambiente - IBAMA, Fundação Nacional do Índio - FUNAI, Comitê de Ética em Pesquisa - CEP, Comissão de Ética no Uso de Animais - CEUA, Comissão Nacional de Energia Nuclear - CNEN, Agência Nacional de Vigilância Sanitária - ANVISA, Comissão Técnica Nacional de Biossegurança - CTNBIO, Comissão Interna de Biossegurança - CIBIO e outras no caso em que a natureza do projeto exigir.
10.3. Declaram o OUTORGADO e a INSTITUIÇÃO SEDE que esta obteve os certificados exigidos pela Legislação de Biossegurança, estando apta a manipular Organismos Geneticamente Modificados e seus derivados, na hipótese de execução de projetos dessa natureza, podendo comprovar mediante declaração que o ateste, quando solicitada.
10.4. Se a execução do projeto vier a ser obstada ou inviabilizada pelo descumprimento das obrigações aludidas nesta cláusula, e não havendo prévia anuência da OUTORGANTE, caberá à INSTITUIÇÃO SEDE ressarcir à OUTORGANTE a totalidade dos recursos concedidos para a execução do projeto, sob pena de ser acionado administrativa e/ou judicialmente pela OUTORGANTE para devolução dos recursos recebidos, devidamente corrigidos pelos índices legais em vigor e com incidência das demais sanções legais (juros, honorários advocatícios e custas judiciais).

CLÁUSULA DÉCIMA PRIMEIRA - DO COMPROMISSO DE OBSERVÂNCIA DA LEGISLAÇÃO RELATIVA À SEGURANÇA:

11.1. Declaram o OUTORGADO e a INSTITUIÇÃO SEDE que é de sua exclusiva responsabilidade solicitar, obter, possuir e demonstrar, quando solicitado pela OUTORGANTE, todos os alvarás, licenças e demais autorizações exigidas por lei para o funcionamento da INSTITUIÇÃO SEDE, quando assim for exigido.
11.2. Declaram o OUTORGADO e a INSTITUIÇÃO SEDE que esta possui os equipamentos de segurança necessários ao seu funcionamento e à execução do projeto em suas dependências, os quais deverão atender às normas técnicas e às exigências dos órgãos de controle e fiscalização.

CLÁUSULA DÉCIMA SEGUNDA - DO COMPROMISSO DE APOIO INSTITUCIONAL DA INSTITUIÇÃO SEDE DO PROJETO:

12.1. A INSTITUIÇÃO SEDE do projeto especificado no preâmbulo garante todo o apoio institucional necessário para sua realização, segundo informação prévia feita por escrito pelo OUTORGADO, constante do Anexo II deste Termo de Outorga.
12.2. Em particular, será garantida ao OUTORGADO e à equipe constante do projeto aprovado pela OUTORGANTE permissão de uso de todas as instalações (laboratórios, rede de computação, biblioteca, base de dados etc.) e acesso a todos os serviços técnicos (de laboratório, de oficinas, administrativo, de compras e importações etc.) disponíveis na Instituição e relevantes para sua execução.
12.3. A INSTITUIÇÃO SEDE se compromete a dar todo o seu apoio institucional para garantir e facilitar o acesso aos equipamentos adquiridos pela OUTORGANTE a pesquisadores de Instituições do Estado de São Paulo e de fora, para fins de projetos de pesquisa científica qualificados.
12.4. A INSTITUIÇÃO SEDE do projeto assume o compromisso de Aceite de Cessão de Uso e/ou Aceite de

Doação dos Equipamentos e Materiais Permanentes adquiridos com recursos do projeto, devendo garantir: o acesso a estes pelo OUTORGADO e pela equipe do projeto, a manutenção em bom estado e a contratação de seguro para proteção dos equipamentos e materiais durante a vigência do projeto ou pelo período especificado no Termo de Aceite de Cessão de Uso e/ou Aceite de Doação, bem como de suas eventuais prorrogações, por pelo menos 10 anos após o término do projeto no caso de haver doação, exceto quando acordado diferentemente com a autorização da OUTORGANTE.

12.4.1. Em caso de sinistro (roubo, furto, avaria ou outro) nas dependências da INSTITUIÇÃO SEDE envolvendo equipamentos destinados à execução do projeto de pesquisa, a INSTITUIÇÃO SEDE deverá tomar todas as medidas administrativas e judiciais para apurar a ocorrência.

12.4.2. Caso os equipamentos sinistrados não tenham sido segurados pela INSTITUIÇÃO SEDE, eventuais custos de reparo dos danos ou de reposição do equipamento serão suportados exclusivamente pela INSTITUIÇÃO SEDE.

12.5. Em caso de falta ou impedimento do OUTORGADO, cabe à INSTITUIÇÃO SEDE notificar imediatamente a OUTORGANTE.

CLÁUSULA DÉCIMA TERCEIRA - DO COMPROMISSO DE OBSERVÂNCIA DO CÓDIGO DE BOAS PRÁTICAS CIENTÍFICAS DA OUTORGANTE:

13.1. Declaram o OUTORGADO e a INSTITUIÇÃO SEDE estar cientes das diretrizes constantes do Código de Boas Práticas Científicas da FAPESP e que se comprometem a respeitá-las.

13.2. Declara a INSTITUIÇÃO SEDE que se compromete a incluir em seu organograma um ou mais órgãos especificamente encarregados da promoção da cultura de integridade ética da pesquisa entre seus pesquisadores e estudantes (mediante a manutenção de programas regulares de educação, disseminação, aconselhamento e treinamento), assim como da prevenção, investigação e punição das más condutas em pesquisa que ocorram em seu âmbito.

CLÁUSULA DÉCIMA QUARTA - DAS ALTERAÇÕES DA CONCESSÃO DO AUXÍLIO E ADITAMENTOS AO TERMO DE OUTORGA:

14.1. Quaisquer alterações no estabelecido neste Termo de Outorga só poderão ser implementadas mediante autorização expressa da OUTORGANTE, formalizada por meio de Termo Aditivo a este Termo de Outorga.

14.2. Ordinariamente e por circunstâncias imprevisíveis, solicitações de Aditivos a este Termo de Outorga para suplementação de recursos ou para alteração do prazo de vigência do Auxílio só serão analisadas pela OUTORGANTE desde que apresentadas juntamente com um Relatório Científico.

14.2.1. As solicitações de Aditivos para extensão do prazo de vigência do Auxílio só serão analisadas pela OUTORGANTE quando encaminhadas com pelo menos 60 dias antes da data final da vigência inicialmente aprovada.

14.2.1.1. A vigência do projeto de que trata o presente Termo de Outorga só poderá ser prorrogada pelo prazo correspondente a até 25% (vinte e cinco por cento) do prazo inicial de vigência.

14.3. Solicitações de Aditivos para prorrogações de bolsas, eventualmente concedidas como item de orçamento

do Auxílio poderão ser analisadas, desde que enviadas juntamente com o Relatório Científico do Auxílio e acompanhadas do Relatório Individual sintético das atividades desenvolvidas pelo bolsista no período e do Plano de Atividades do bolsista para o próximo período.

CLÁUSULA DÉCIMA QUINTA - DAS DISPOSIÇÕES GERAIS:

15.1. O OUTORGADO declara que aceita, sem restrições, este Auxílio, tal como concedido, e se responsabiliza pelo fiel cumprimento do presente Termo de Outorga em todos os seus itens, cláusulas e condições, e que concorda com qualquer fiscalização que a OUTORGANTE julgar conveniente proceder, de acordo com o inciso III do artigo 3º da Lei nº 5.918, de 18 de outubro de 1960.

15.2. O OUTORGADO declara que tem plenas condições de realizar as atividades previstas no projeto de pesquisa e que envidará todos os esforços para que seus objetivos sejam atingidos.

15.2.1. Declara o OUTORGADO também que deu ciência por escrito às instâncias competentes da INSTITUIÇÃO SEDE das necessidades infraestruturais e do apoio institucional indispensável para o bom andamento do projeto e que recebeu destas a aprovação quanto à garantia deste apoio, conforme o documento constante do Anexo II deste Termo de Outorga.

15.3. Em caso de abandono do projeto, sem prévia autorização da OUTORGANTE, o OUTORGADO se compromete a restituir à OUTORGANTE, imediatamente, todos os recursos concedidos para a execução do projeto, sob pena de ser acionado administrativa e/ou judicialmente pela OUTORGANTE para a devolução dos recursos recebidos, devidamente corrigidos pelos índices legais em vigor e com incidência das demais sanções legais (juros, honorários advocatícios e custas judiciais).

15.4. A violação de qualquer das cláusulas do presente Termo de Outorga importará em suspensão do Auxílio concedido e/ou retirada dos materiais adquiridos.

15.5. As comunicações e solicitações referentes a este Termo de Outorga devem ser apresentadas conforme as instruções para comunicação sobre Auxílios em andamento, disponíveis no portal da FAPESP (www.fapesp.br).

15.6. Fica eleito o foro da Fazenda Pública da Comarca de São Paulo como competente para resolução de eventuais conflitos.

15.7. Após sua assinatura, o presente Termo entrará em vigor na data indicada para início do projeto.

15.8. Integram o presente Termo de Outorga, para todos os efeitos legais, as instruções constantes dos Anexos:

15.8.1. Anexo I: Relação dos Benefícios e Materiais Concedidos.

15.8.2. Anexo II: Informação aprovada pela Instituição Sede sobre a infraestrutura Institucional.

15.8.3. Anexo III: Manual de Instruções para Uso dos Recursos e Prestação de Contas de Auxílios e de Reserva Técnica concedidos pela FAPESP (Portaria PR nº 03/2011).

15.8.4. Anexo IV: Política para Propriedade Intelectual da FAPESP (Portaria PR nº 04/2011).

15.8.5. Anexo V: Normas para utilização dos recursos da Reserva Técnica concedidos pela FAPESP (Portaria PR nº 06/2011).

15.8.6. Anexo VI: Código de Boas Práticas Científicas da Outorgante (Deliberação do CTA nº 02/2013).

CLÁUSULA DÉCIMA SEXTA - DAS DECLARAÇÕES DE CIÊNCIA SOBRE O DISPOSTO NESTE TERMO DE

OUTORGA:

16.1. O Dirigente da INSTITUIÇÃO SEDE declara estar ciente de que o descumprimento de quaisquer cláusulas deste Termo de Outorga poderá prejudicar o andamento de futuras solicitações apresentadas à OUTORGANTE por pesquisadores associados à INSTITUIÇÃO SEDE. Declara ainda que leu e teve ciência das condições do presente Termo de Outorga, mediante identificação legível (nome e cargo) e assinatura a seguir.

16.2. O OUTORGADO declara estar ciente de que o descumprimento de quaisquer cláusulas deste Termo de Outorga poderá prejudicar o andamento de futuras solicitações por ele apresentadas à OUTORGANTE. Declara ainda que leu e teve ciência das condições do presente Termo de Outorga, mediante assinatura a seguir.

São Paulo, 1 de Abril de 2018.

Outorgado ____

Pela Instituição Sede ____

Pró-Reitoria de Pós-Graduação, Pesquisa e Extensão/PRPGPE/UNICID

Outorgante

______________________	______________________
Andreia Alves Neves	Fernando Dias Menezes de Almeida
Por Procuração	Diretor Administrativo

Para uso exclusivo da FAPESP
Processo: 2016/24217-7
Data impressão: 12/04/2018 12:23:46
Folha: ________________
Volume: _______________
Rubrica: _______________

ANEXO I: Relação dos Benefícios e Materiais Concedidos

Capital

Material Permanente

1 Microcomputador portátil e componentes

Custeio

Material de Consumo

Não houve concessão para esta alínea.

Serviços de Terceiros

Materiais e/ou serviços estritamente necessários para a realização deste projeto.

Despesas de Transporte

Não houve concessão para esta alínea.

Reserva Técnica - Benefícios Complementares

Utilização conforme normas vigentes.

Pesquisador	Função	Início	Término	Possui BC
Bruno Tirotti Saragiotto	Pesq.Responsável	01/04/2018	31/03/2022	Sim

Reserva Técnica - Custo de Infraestrutura Direta do Projeto

Utilização conforme normas vigentes.

Infraestrutura

Não houve concessão para esta alínea.

Diárias

Não houve concessão para esta alínea.

Concessão de Quotas de Bolsas

Quota de Bolsa Orçamentária:
ver campo "Observações"

Modalidade / Categoria	Carga Horária	Duração (Meses)	Quantidade Concedida
JP		24	1
DD		48	1

Concessão Total	
Parte em Reais: estão incluídos neste valor os recursos concedidos ou convertidos para real	R$ 267.683,35

Observações

- Foi concedida quota de Bolsa orçamentária, podendo haver dedução de bolsa anterior quando a regra se aplicar.
  O candidato selecionado somente poderá iniciar suas atividades no projeto após a aprovação de seu cadastro, que deve ser submetido via SAGe, conforme Instrução Normativa específica para cada modalidade, constante no site da FAPESP em www.fapesp.br/bolsas/bolsasconcedidasemauxilios.
  Não serão feitos pagamentos ou reembolsos referentes a bolsistas fora do respectivo processo SAGe.
  A vigência da bolsa não poderá ultrapassar a do auxílio.
