# Supplementary material for: Evaluation of the efficacy of an internet-based pain education and exercise program for chronic musculoskeletal pain in comparison with online self-management booklet: a protocol of a randomised controlled trial with assessor-blinded, 12-month follow-up, and economic evaluation
Source: BMC Musculoskelet Disord. 2020 Jun 26;21:404. doi: 10.1186/s12891-020-03423-x (PMC7320555; doi:10.1186/s12891-020-03423-x)
Supplement: Supplementary file 1 — Additional file 1. [file Peer-review process FAPESP_translatedR1.pdf › Peer-review process FAPESP_translatedR1.pdf]

FUNDAÇÃO DE AMPARO À PESQUISA DO ESTADO DE SÃO PAULO

APPLICATION FORM FOR RECONSIDERATION OF FELLOWSHIPS AND AID (Exclusive use by the SAGe system)

PROCCESS: **2016/24217-7**
RESEARCHER: Bruno Tirotti Saragiotto
SUPERVISOR (IF ANY):

1- INDICATE WHICH DOCUMENTS HAVE BEEN ADDED TO THE CURRENT PROPOSAL

☒ RESEARCH PROJECT REVISED	☒ CV	☐ SCHOOL RECORDS
☒ OTHER: (1) Master's academic record; (2) Reformulated budget; (3) Budget of suppliers; (4) Reformulated DD scholarship work plan; (5) Work plan for TT-4 bag reformulated; (6) Letter from the coordinator of the linked institution for accreditation in the program; (7) Justification of the reformulated budget items; (8) Proof of doctoral completion.		

2- ANSWER BELOW, POINT BY POINT, EACH QUESTION RAISED IN ADVISORY OPINIONS, INDICATING THE CORRESPONDING MODIFICATION TO THE PROPOSAL.
IMPORTANT: If there are changes in the research project motivated by advice, they must be incorporated and highlighted in a reformulated research project, to be submitted to FAPESP with this request for reconsideration.

Dear FAPESP and Partners,

I would like to thank you for the opportunity to request the Young Researchers in Emerging Centers Program (JP-FAPESP), and for the evaluation carried out by the reviewers of the research project entitled "The effectiveness of a tele-rehabilitation program for the treatment of chronic musculoskeletal pain: Program e-Rehab ", case number: 2016 / 24217-7.

After a thorough reflection on the opinion, a point-by-point review of the comments made by the referees can be found below. Based on the changes and adjustments made to the research project (carried out in accordance with the suggestions and comments of the referees), I believe that the project is now suitable for the proposal. Therefore, I would like to request a reconsideration request for this project.

The changes made to the research project motivated by advisory opinions are highlighted in yellow in the reformulated project, as requested by FAPESP.

Peer-to-peer review

<u>1st Advisory Opinion (Partner 1)</u>

GENERAL ANALYSIS OF THE PROPOSAL FAPESP calls "Proposal" the set of four parts to be analyzed, composed of: 1. Academic History of the Researcher (a) Responsible; 2. Research Project; 3. Conditions offered by the Institution where the project will be developed; and, 4. Budget. Please complete this item after completing the other items on this form.

The researcher presents an Academic History with only two subjects during the entire PhD period.
Perhaps this is the case in Australia where the PhD was held. It was not possible to evaluate the Master due to the lack of documentation.

The research project is relevant and interesting.
The institution where the project will be developed is in full condition since the study will be carried out mainly by means of tele-rehabilitation.
The amounts for requesting financing were presented, but without due budgets.

Answer: Thank you very much for the opinion and suggestions. Actually for the doctorate at the University of Sydney only 10 credits are required, which is usually accomplished with 2 or 3 courses (depending on the amount of credits for each course). The two subjects I took were chosen together with my doctoral advisor. Other subjects are possible to be taken, but at an extra cost and my scholarship did not cover any of these extra costs. I also added as a supplementary document the academic record of the master with the disciplines fulfilled so that the referee can also evaluate this item.

Please review the RESEARCHER'S RESPONSIBLE ACADEMIC HISTORY of the Project considering the items below:

Analyze the quality and regularity of scientific and / or technological production. Important elements for this analysis are: list of publications in journals with selective editorial policy; books or book chapters; patents in which he appears as an inventor; other intellectual property instruments; research results effectively transferred and adopted by companies or the government; and other information that may be relevant.

The researcher presents a good and regular publication. Looking at Lattes, you can see around 50 publications. However, several of these publications are editorials or letters to the editor and this made it difficult to analyze the publications. Editorials on the TIDier Checklist, for example, are presented as complete articles about 15 times since the checklist has been published in different journals.

Analyzing the 10 articles selected by the researcher in the Súmula Curricular, the researcher is the first author of 5 (with 3 systematic reviews) and an editorial, the second author in 2, and the third author in 2. The publications are in a high impact magazine in the area .

Answer: Thanks for the review. I currently have 53 publications (16 as the first author), 16 of which are editorials on the TIDieR checklist, 1 editorial on chronic pain in the British Journal of Sports Medicine and 1 response to a letter to the editor we received (published in JOSPT). It is important to remember that editorials are generally published from an invitation by the magazine, which demonstrates the impact of my research in the area. In addition, in the Lattes curriculum there is no option to differentiate editorials or letters to the editor from original articles and it is also not possible to add TIDieR editorials as a single record since this was an initiative involving 16 different physiotherapy scientific journals and different authors for each editorial. The success of this initiative reflects the impact of my research and the group I am part of and my collaborations. More than 20 scientific journals around the world have subscribed to the TIDieR checklist (16 editorials published so far), which should result in more reproducible studies in the area in the near future (that is, clinicians having access to more details of the interventions performed in the research, being able to apply it with greater precision and safety to their patients), in addition to less waste of resources in the area. Avoiding the waste of money, time and resources in research was pointed out as a priority for researchers in a special edition of The Lancet published in 2015.

In relation to the 10 articles selected in the curriculum summary, I made this selection based on the impact and citations of the studies. However, at this moment, the curriculum summary was changed and updated to better reflect my contribution and impact in the area.

Analyze the experience demonstrated in research projects related to the theme of the proposed project.

The researcher has experience in the area of low back pain. However, telereabilitation appears to be innovative.

Answer: Thanks for the comment. I worked with sports injuries during my master's degree following the line of research of my advisor at the time. During my doctorate, I worked particularly with back pain and chronic pain, being the line of research of my doctoral advisor. I believe that the aim of the Young Researchers in Emerging Centers Program is to enable the creation of job opportunities for researchers in the stage of professional affirmation so that they can establish new research centers in emerging centers. My professional

objective with this program then is to establish my own line of research for the next years in distance interventions, such as tele-rehabilitation. In parallel with my doctorate, I had the opportunity to be involved in some projects with distance interventions that are being developed at the University of Newcastle, at the "Center for Pain, Health and Lifestyle" (www.centrephl.org), such as a review systematic that I am currently leading on smoking interventions to improve chronic pain involving tele-rehabilitation. These projects have been very important for me to gain experience with this intervention model. In addition, during the review period of this application, the systematic reviews that were attached to this project were approved as 3 Cochrane reviews (telerehabilitation for low back pain, cervical pain and knee and hip osteoarthritis) that will be led by me, which demonstrates the potential impact of this line of research.

Analyze the candidate's international research experience (short and long internships, participation in collaborations), considering the quality of the published results.

Candidate did a Full Doctorate in Australia with CNPq scholarship.

Answer: nothing to declare.

Conclusion on the analysis of the Academic History of the Researcher Responsible for the Project.

[ ] Great

[X] Very good

[ ]Good

[ ] Regular

[] With serious deficiencies

Answer: Nothing to declare.

Conclusion on the Institutional Conditions where the Project will be carried out

[ ] Great

[X] Very good

[ ]Good

[ ] Regular

[] With serious deficiencies

Answer: Nothing to declare.

Please review the RESEARCH PROJECT following the script below.

Analyze the definition, relevance, originality of the objectives and the importance of the intended contribution to the area of knowledge in which the proposed project is inserted.

The project is original and relevant. The objectives are well defined and will contribute to the literature on low back pain. A clinical trial and two systematic reviews will be carried out on telereabilitation in low back pain and osteoarthritis.

Answer: Thanks for the comment. The project aims to include the main chronic pain conditions, so the main study (clinical trial) will include participants with spinal pain and knee and hip osteoarthritis (the most

common chronic pain in the world). Systematic reviews have been updated in this new version of the project to 3 Cochrane reviews on tele-rehabilitation for low back pain, cervical pain and hip and knee osteoarthritis. More details of the methods of these reviews can be found in Appendices 1, 2 and 3.

Analyze the scientific basis and the methods used.

The project is well-founded on the basis of the available literature. Regarding the methods that will be used there are some doubts:

how will adherence be controlled? I understand that the idea is to call and send messages, but we can never be sure what people are really doing and this should be considered in the study.

I believe that the group that will receive the intervention will improve when compared to the control group. Shouldn't there be another group receiving conventional therapy, or should that group receive SMS, for example, warning about the time left to be attended (perhaps one SMS per month)?

Is 1 point of difference in VAS between groups clinically relevant?

Answer: Thanks for the comment. Indeed, I believe that these are important points and that they must be taken into account. Below is the answer to each of your comments.

a) Adherence to treatment has been reported as one of the main barriers in distance treatment programs. One way to try to overcome this problem was to include follow-up with text messages and weekly calls that aim to try to keep patients encouraged to run the program. This is a strategy that has already been shown to be efficient and suggested by associate researcher Blake Dear, who will contribute to this project as an expert in remote interventions. To monitor patient compliance in this project, we will distribute diaries to patients (in the baseline assessment) so that they can also register or have their own control of compliance with the program. This is described in the project, in the subsection of the intervention description ("Adherence to the program will be monitored by a daily log that the patient will bring to the follow-up at the end of the treatment").

b) Thanks for the suggestion. The idea of adding a group receiving minimal intervention or conventional therapy is very interesting. However, the addition of a third group in this study would reduce the statistical power and precision of the study, unless we increase the sample size to at least double (maintaining a power of 90%), which could make the study unfeasible at this time. . As this project is a proposal to start a line of research in which we will highly appreciate the feasibility, we believe that at the moment the best option is to keep both groups. In addition, the proposed budget has already been considered "overestimated" by two of the reviewers and we believe that adding a group would bring even more costs to this project.

c) Thanks for the comment. After a careful review of this point with the researchers involved in the project, we decided to recalculate the sample size based on a recent clinical trial published in the New England Journal of Medicine (Mathieson 2017). Therefore, we considered a difference between the groups of 1.5 points in the VAS, which provides a representative sample for the study (160 subjects). Increasing the difference between groups for the sample calculation (for example, 2 points), would drastically decrease the sample of this study, which has been a limiting factor for the statistical power of clinical trials in the area.

The full description of the sample calculation has been added to the section "3.8.1 Sample Size Calculation", and is described below:

"We determined that a minimum of 160 individuals (80 per group) would be required to provide the trial with 90% power to detect a between-group difference of 1.5 point in a 0 to 10 Pain Numerical Rating Scale, with a standard deviation of 2.75 points and two-sided alpha level of 0.05. The estimated sample size would also allow for a withdrawal rate of 15%. "

reference:

Mathieson S, Maher CG, McLachlan AJ. Trial of Pregabalin for Acute and Chronic Sciatica. New Engl J Med. 2017; 376 (12): 1111-1120.

Is the proposed deadline adequate for the development of the project?

[X] Yes

[ ] Not

Answer: Nothing to declare.

Analyze the scholarships requested as an integral part of the project (Scientific Initiation (IC), Technical Training (TT), Master's (MS), Direct Doctorate (DD), Pedagogical Improvement (EP) and Scientific Journalism (JC)). For the Young Researcher Project, FAPESP may grant, as part of the program budget, IC, TT, MS, DD, EP and JC scholarships, according to the rules described in: http://www.fapesp.br/bolsas/bolsasconcedidasemauxilios, by evaluating the project and the Activity Plan for each scholarship requested. If approved, the scholarship holder must be selected by the project applicant, according to FAPESP rules, available at the addresses mentioned in each case:

Analyze the adequacy of the quantity and Activity Plans proposed for Technical Training scholarship holders (http://www.fapesp.br/materia.php?data{id_materia/201=3171)

The proposal requests 1 TT3 and 1 TT4. I consider it appropriate.

Answer: Nothing to declare.

Analyze the appropriateness of the quantity and Activity Plans proposed for Scientific Initiation fellows (http://www.fapesp.br/materia.php?data{id_materia/201=2889)

AT

Answer: Nothing to declare.

Analyze the adequacy of the quantity and Activity Plans proposed for Master's scholarship holders (http://www.fapesp.br/8685)

AT

Answer: Nothing to declare.

Analyze the appropriateness of the quantity and Activity Plans proposed for Direct Doctoral fellows (http://www.fapesp.br/materia/4789)

It was not clear what exactly the PhD project would be. I understand that the PhD student would participate in the entire project, but what would the PhD specifically be?

Answer: The work plan for the doctoral scholarship is submitted together with the project as additional material. The doctorate will consist mainly of three studies. Two Cochrane systematic reviews, the pilot study and the randomized cynical trial. The systematic review will aim to assess the effectiveness of tele-rehabilitation for patients with low back pain and neck pain, and the pilot study and clinical trial will test the tele-rehabilitation program (main project). The student will be responsible for these studies and his doctoral thesis may contain additional parallel studies as the student evolves in the management of these studies. The work plan for the doctorate has been updated to clarify this information.

Analyze the appropriateness of the quantity and Activity Plans proposed for Scholarships for Pedagogical Improvement (EP). (http://www.fapesp.br/46)

AT

Answer: Nothing to declare.

Analyze the adequacy of the quantity and Activity Plans proposed for Scientific Journalism (JC) fellows.

(http://www.fapesp.br/jornalismocientifico)

AT

Answer: Nothing to declare.

Conclusion on the Analysis of the Research Project presented

[ ] Great.

[] Very good.

[X] Very good, with some easily remedied deficiencies.

[ ]Good.

[] Good with disabilities.

[ ] Regular.

[] With serious deficiencies.

Answer: Thanks for the analysis. I hope that the responses above to the comments may have cleared up any doubts regarding the project and remedied the deficiencies pointed out by the referee.

Please review the PROPOSED BUDGET for:

Need for Equipment and Permanent Materials requested to carry out the project, also taking into account the infrastructure already available at the institution and the ability of the applicant and his team to use them.

Does permanent equipment and materials, national or imported, with a value greater than R $ 20,000.00 have your need justified in the proposal?

Opinion, in each case, on the validity of the justification presented in the item above

There is no request for permanent material over R $ 20,000.00

Answer: Nothing to declare.

Are there permanent equipment and materials for which FAPESP should or can request the establishment of a system of access to third parties not involved in this Project?

Not

Answer: Nothing to declare.

Analyze the need for consumables requested for the project.

There is no request for Consumables.

Answer: Nothing to declare.

Analyze the need and adequacy of Third Party Services for the project.

Are Third Party Services only of a technical and occasional nature, as required by FAPESP rules?

The requested third party services are fundamental for the development of the research.

Answer: Nothing to declare.

In any case, is it reasonable to expect that the requested service should be provided by the institution hosting the project, in return for support from FAPESP?

Perhaps the Dropbox subscription and Disclosure payment on Facebook.

Answer: Thanks for the suggestion. These items have been removed from the budget since the linked institution will offer support in the dissemination of the study for recruitment and storage of data.

Suggest alternative values for the budget (Should be excluded from the budget: salaries of any kind, third-party services that are not of a technical and eventual nature, civil works, acquisition of publications, trips except for field research, material and administrative services).

National permanent material.

No budget was presented.

Answer: Nothing to declare.

Imported permanent material.

No budget was presented.

Answer: Nothing to declare.

National consumption material.

AT

Answer: Nothing to declare.

Imported consumables.

AT

Answer: Nothing to declare.

Third party services in the country.

No budget was presented.

Answer: Nothing to declare.

Third party services abroad.

No budget was presented.

Answer: Nothing to declare.

Transport expenses in the country.

AT

Answer: Nothing to declare.

Transport expenses abroad.

AT

Answer: Nothing to declare.

Daily rates in the country.

AT

Answer: Nothing to declare.

Daily rates abroad.

AT

Answer: Nothing to declare.

Various national.

AT

Answer: Nothing to declare.

Various imported.

AT

Answer: Nothing to declare.

Grand total national currency.

AT

Answer: Nothing to declare.

Grand total foreign currency.

AT

Answer: Nothing to declare.

Scholarships Based on the analysis carried out in the previous item, suggest the amount of IC, TT, MS, DD, EP and JC scholarships.

0 Scientific Initiation (IC) 0 Technical Training I (TTI) 0 Technical Training II (TTII) 1 Technical Training III (TTIII) 1 Technical Training IV (TTIV) 0 Technical Training IVa (TTIVa) 0 Technical Training V (TTV) 0 Master (MS) 1 Direct PhD (DD) 0 Pedagogical Improvement (EP) 0 Scientific Journalism

Answer: Nothing to declare.

Conclusion on the proposed budget

We are a family owned and operated business.

[] Adequate [X] May be Adequate if changes are suggested above [] Inappropriate

Answer: The budget was revised and updated according to the referee's suggestions.

NOTED DISABILITIES (If any item is checked, the reasons must be indicated in the corresponding table on the form)

About the Researcher's Academic Background:

[] Scientific or technological production that does not attest to significant performance of the research activity.

[] Insufficient experience in the research area in which the project is inserted, which may compromise its viability.

[] The scientific or technological production resulting from previous aid is unsatisfactory.

[] The applicant does not meet the requirements of the program rules.

Answer: Nothing to declare.

About the Institution and the Research Group:

[] The Institution's institutional and physical infrastructure is insufficient.

[] Lack of institutional commitment to the success of the proposal.

[] The proposal will not contribute to the nucleation of new research groups.

[] The proposal will not contribute to the decentralization of the state research system.

[] Center with a tradition of research in the area in which the project is inserted, with no exceptional circumstances in the light of the program rules.

Answer: Nothing to declare.

About the project:

[] Project with ill-defined, excessive or incongruous objectives.

[] Project with excessively limited objectives.

[] Unoriginal design.

[] Insignificant contribution to the area of knowledge.

[X] Poor scientific foundation / inadequate methodology.

[] Feasibility of questionable execution.

[] Excessive cost in view of the expected scientific or technological contribution or the probability of success of the project.

[] Activity plans for TT fellows are unsatisfactory.

[] The activity plans for IC fellows are unsatisfactory.

[] Activity plans for MS fellows are unsatisfactory.

[] Activity plans for DD fellows are unsatisfactory.

[] The activity plans for EP fellows are unsatisfactory.

[] Activity plans for JC fellows are unsatisfactory.

[] The number of TT fellowships requested is inadequate.

[] The number of IC fellowships requested is inadequate.

[] The number of MS scholarship holders requested is inadequate.

[] The number of DD scholarship holders requested is inadequate.

[] The number of EP fellows requested is inadequate.

[] The number of JC fellows requested is inadequate.

[] Inadequate term.

Answer: I believe that the answers to the questions above, as well as the changes and updates in the project, have solved any doubts of the referee in relation to the methodology and quality of the study. The study design is a randomized controlled study to test the effectiveness of a tele-rehabilitation program, which we believe is the best study design for this purpose. Among the associate respondents, three of them are researchers with extensive experience in clinical trials who have reviewed the study plan and who have published their clinical trials in renowned scientific journals such as the New England Journal of Medicine, The Lancet and Pain.

About the Proposed Budget:

[] Items insufficiently justified.

[] Overestimated budget.

Answer: Nothing to declare.

Others (explain):

No budgets have been submitted.

Answer: I believe there was some mistake, since the budget was submitted together with the project and analyzed by the other reviewers.

Opinion 2nd Advisory

GENERAL ANALYSIS OF THE PROPOSAL FAPESP calls "Proposal" the set of four parts to be analyzed, composed of: 1. Academic History of the Researcher (a) Responsible; 2. Research Project; 3. Conditions offered by the Institution where the project will be developed; and, 4. Budget. Please complete this item after completing the other items on this form.

The applicant's scientific production is significant with 50 articles published in selective editorial policy journals. You have no published books or book chapters or other results. The research project is interesting but contains some inconsistencies. The Institution has a tradition in research. The project does not characterize a new line of research since several of the Institution's professors are already carrying out research on this topic. I suggest that the budget be revised as the figures are very high.

Answer: Thank you for reviewing the proposal. Below are the detailed answers to the referee's questions regarding school history, the innovation of the research line, the project and the budget.

Please review the RESEARCHER'S RESPONSIBLE ACADEMIC HISTORY of the Project considering the items below:

Analyze the quality and regularity of scientific and / or technological production. Important elements for this analysis are: list of publications in journals with selective editorial policy; books or book chapters; patents in which he appears as an inventor; other intellectual property instruments; research results effectively transferred and adopted by companies or the government; and other information that may be

relevant.

The applicant's scientific production is significant with 50 articles published in selective editorial policy journals, 28 of which were published in 2016. There are no published books or book chapters or other results.

Answer: Nothing to declare.

Analyze the experience demonstrated in research projects related to the theme of the proposed project.

The doctorate was with the theme of low back pain and therefore has a good experience.

Answer: Nothing to declare.

Analyze the candidate's international research experience (short and long internships, participation in collaborations), considering the quality of the published results.

Good experience because the applicant is doing his PhD in Australia

Answer: Nothing to declare.

When appropriate, analyze the results obtained by the applicant with previous financing from FAPESP or other agencies, including scholarships.

Not applicable

Answer: Nothing to declare.

Analyze other items that make up the curriculum summary.

Not applicable

Answer: Nothing to declare.

According to the rules of the program, the applicant must be a researcher still in the stage of professional affirmation, but already with a very good production for the stage of his career. In particular, the candidate must be fully capable of coordinating research projects independently and implementing new lines of research. Does the applicant fulfill these requirements?

[X] Yes

[ ] Not

Justify:

It partially meets the requirements, as it has no experience in coordinating research projects and the proposed line of research is already being developed at the Institution where it intends to develop the project.

Answer: Thanks for the comment. The curriculum summary was modified and updated in order to better reflect my academic experience and the impact of the research that I have been developing. I submitted my doctoral thesis at the University of Sydney in March 2017 and received a PhD title in May 2017 without any consideration (review) from the 3 examiners (which puts me among 1% of students across the University of Sydney). In this year of 2017 one of my doctoral studies, in which I am the first author, received a mention from the journal Annals of Internal Medicine (impact factor: 17.2) as one of the 8 most important articles published in 2016 regarding quality and impact on global health, which exemplifies the impact around the world of the research that I've been developing. During my doctorate, I was also approved as an associate editor of the Cochrane Back and Neck group. In addition, I lead two initiatives that demonstrate my commitment and participation in the research area. The first initiative is PEDrinho (https://www.facebook.com/PhysiotherapyEvidenceDatabase.PEDrinho/), a blog on social networks that aims

to promote high quality clinical evidence and updates to the PEDro database in Portuguese (5,000 followers). I am one of the creators of this blog that is part of PEDro (the largest database of physiotherapy in the world), where I worked throughout my doctorate. The second initiative is ICECReam (International Collaboration for Early Career Researchers - www.theicecream.org), a collaboration network for young researchers around the world. This network of collaborations was created in 2010 and today it is led by me and two colleagues. This initiative provided me with several opportunities, as well as collaborations and workshops for young researchers at important conferences in the area, such as the WCPT (World Confederation for Physical Therapy Congress) and the Low Back Pain Forum.

During the past few years, in parallel with my doctorate, I had the opportunity to lead several studies with different methodologies, including cohort, cross-sectional, qualitative, systematic reviews and methodological evaluations. In addition, since before the end of my doctorate, I have also had the opportunity to work as a research assistant in a clinical trial on low back pain prevention (Stevens 2016), in which I was able to participate in the preparation, recruitment and monitoring of the participants in this study. . Being part of a large-scale clinical trial team (n = 1,482) has been important for me to understand the practical challenges of a clinical trial and to apply this learning to my future project. The Young Researcher project would then be an opportunity to lead and conduct a randomized controlled study, contributing to the development of my own line of research. I believe that I am able to lead this type of study due to my academic experience to date and the training in clinical research and clinical trials that I had at one of the best research institutions in the world, mainly in Physiotherapy (The University of Sydney was ranked as number 1 by the "Quacquarelli Symonds World University Ranking"). As an example, I can say that the clinical trials carried out in the research group that I am part of and that are carried out by the researchers I currently have a partnership with, have been published in important journals, such as the New England Journal of Medicine and The Lancet. I also have complete training on the PEDro scale for clinical trials (more than 1,000 methodological quality analyzes performed by me for the PEDro database), which demonstrates my experience with the methodology. In addition, to complement my experience, all researchers associated with this Young Researcher project have experience with clinical trials in Brazil and abroad, which provides security for the project's viability.

My professional objective for the coming years is to develop a line of research with remote interventions for different health conditions, not just limiting it to the condition of chronic pain. This line of research has not been developed at the institution to which this project is linked and, at the moment, there seems to be no line of research on remote interventions in Brazil. I believe that this line of research is extremely important for our country, especially in the city of São Paulo, where the health service has a very high demand. This type of intervention is growing exponentially in the world. According to a report in the Wall Street Journal (USA), more than 15 million Americans received some type of remote intervention through technologies in 2016 and this number was estimated to increase by 30% in 2017. According to health technology expert Larry Jones , it is estimated that in 2018 more than 90% of large American companies (+1,000 employees) are expected to offer tele-medicine services (distance medical services) to their employees. In addition, the American College of Physicians (ACP), in its latest recommendation on the subject, strongly recommends the use of tele-medicine in clinical practice to amplify access to health and decrease health costs. So I believe that this line of research is very promising to be developed in Brazil and can bring numerous benefits to the country's health system.

The proposed project focuses on chronic pain, due to my experience in the area, in addition to the experience of associate researchers in this area. As my line of research consolidates in the future, I intend to continue with other chronic conditions that also need attention (for example: diabetes and fibromyalgia). I currently have a collaboration with the "Center for Pain, Health and Lifestyle (www.centrephl.org)", a group located at the University of Sydney and at the University of Newcastle, where I have the opportunity to work on projects with remote interventions for different conditions. health, such as a systematic review I am leading on smoking control interventions on the impact of chronic pain, which includes distance interventions. There are professors at the linked institution who study low back pain and cervical pain, which will contribute to the development and viability of the line of research that I intend to work with this project.

References:

1. Link to article in the Wall St Journal: https://www.wsj.com/articles/how-telemedicine-is-transforming-health-care-1466993402?mod=itp&mod=djemITP_h

2. Link to Larry Jones article: https://www.linkedin.com/pulse/telehealth-living-up-promise-larry-jones

3. Daniel H, Sulmasy LS, for the ACP Health and Public Policy Committee. Policy recommendations to guide the use of telemedicine in primary care settings: an American College of Physicians position paper. Ann Intern Med 2015; published online 8 Sep, doi: 10.7326 / M15-0498.

4. Stevens ML, Lin CC, Hancock MJ, et al. TOPS: Trial Of Prevention Strategies for low back pain in patients recently recovered from low back pain - study rationale and protocol. BMJ Open 2016; 6: e011492. doi: 10.1136 / bmjopen-2016-011492.

Conclusion on the analysis of the Academic History of the Researcher Responsible for the Project.

[ ] Great

[ ] Very good

[X] Good

[ ] Regular

[] With serious deficiencies

Answer: We hope that the clarifications above and in the new documents attached to the proposal can resolve any doubts regarding the academic record and the capacity of the research team to develop the project, as well as the potential of this new line of research in the institution where the project is linked.

Please review the INSTITUTIONAL CONDITIONS in which the proposal is inserted, following the script below.

Analyze the institution's research tradition. Is the project characterized as a new line of research at the institution with a consolidated tradition in research?

The Institution has a tradition in research. The project does not characterize a new line of research since several of the professors of the Postgraduate Program are already carrying out research with this theme.

Answer: Based on the answer to the previous item, I would like to request reconsideration for this opinion. The proposed line of research has great potential in the city of São Paulo and is characterized as a new line of research at the institution, as well as innovative in Brazil. It is important to make it clear that the line of research is related to tele-rehabilitation, not the health condition, in which, it is known that there are other UNICID researchers working in this area.

Analyze the adequacy of the institutional and physical infrastructure offered by the Institution in which the project will be developed and the institutional commitment to the proposal, according to the program rules.

The Institution expressed itself in favor

Answer: Nothing to declare.

Analyze the effects that support for the proposal may have on the Institution. Note that: 1) The purpose of the Young Researchers in Emerging Centers Program is to enable the creation of adequate job opportunities for young researchers or groups of young researchers with great potential, preferably in emerging research centers, favoring the nucleation of new groups and the decentralization of the state research system. Candidates who intend to create a new line of research in an institution with a consolidated research tradition may also enroll in the program; 2) in the case of an institution with a tradition of research, the proposal will be approved only if: a) It is a candidate with exceptional performance for the stage of his career; b) Special circumstances are identified (for example, it is a proposal to create a new research group in the unit or department) that may justify the support of the

proposal in this program.

1. The candidate is not proposing a new line of research because it is already being developed at the Institution 2. The candidate had a very good performance during his doctorate abroad

Answer: Once again, based on the answers above, I kindly ask that the referee reconsider the analysis of the academic history and the line of research to be developed as a result of this project. The curriculum summary has been updated to better exemplify my contribution to research and impact in the area. I believe, for the reasons mentioned above, that I am able to lead this project and develop this line of research.

Conclusion on the Institutional Conditions where the Project will be carried out

[ ] Great

[ ] Very good

[X] Good

[ ] Regular

[] With serious deficiencies

Answer: Regarding the institution's conditions, UNICID's Graduate Program has a concept 4 at CAPES (the doctoral program started in 2013). Four professors of this program obtained research productivity grants at CNPq in the last 4 years (professors Leonardo Costa, Richard Liebano, Monica Perracinni and Sandra Freitas). The same program was approved in eight regular projects at FAPESP in the last 4 years and 8 universal calls from CNPq in the same period, totaling almost R $ 3 million in support of these two research funding agencies (CAPES and CNPq). In addition, the professors of this institution form editorial boards of national and international scientific journals (such as JOSPT, Physical Therapy, Brazilian Journal of Physiotherapy, among others), which represents the contribution of these teachers to the area. This institution is, therefore, a center that has been developing a lot in recent years and is characterized as an emerging center, which is the objective of the Young Researcher proposal. The approval of this proposal should contribute significantly to the development of this postgraduate program and insert an innovative line of research at the institution and in Brazil on remote interventions for health conditions. In the last forum of the Brazilian Association of Post-Graduation in Physiotherapy, Prof. Dr. Tania Salvini presented the data of physiotherapy in relation to the promotion of funding at FAPESP and UNICID comes in third place, just behind UFSCAR and USP.

There is enormous potential for this new line of research, since the Unified Health System (SUS) in São Paulo is overcrowded and many patients with non-serious conditions should benefit from remote interventions. For example, patients with chronic pain usually take months or even years in lines for treatment in the current Brazilian health system. If this tele-rehabilitation program proves effective for these patients, millions of people with chronic pain will be able to benefit in the future and contribute to reduce the flow of people waiting for treatment in the health system. Thus, the health system will be able to better direct its resources to conditions where it is not possible to use a tele-rehabilitation system, such as chemotherapy, heart disease, acute neurological conditions and emergencies. Previous studies show that for every kilometer away from urban centers, quality and access to health care decrease (Kelly 2016, Jordan 2004). In addition, the World Health Organization (WHO) recognizes access to health as a determining factor for worse health rates.

References:

1. Kelly C, Hulme C, Farragher, T, Clarke G. Are differences in travel time or distance to healthcare for adults in global north countries associated with an impact on health outcomes? A systematic review. BMJ Open. 2016; 6 (11): e013059. doi: 10.1136 / bmjopen-2016-013059.

2. Jordan H, Roderick P, Martin D, Barnett S. Distance, rurality and the need for care: access to health services in South West England. Int J Health Geo. 2004; 3:21. doi: 10.1186 / 1476-072X-3-21.

3. Health Impact Assessment: The Determinants of Health - World Health Organization [available at

http://www.who.int/hia/evidence/doh/en/].

Please review the RESEARCH PROJECT following the script below.

Analyze the definition, relevance, originality of the objectives and the importance of the intended contribution to the area of knowledge in which the proposed project is inserted.

The project is interesting, but it has some inconsistencies. It points out that there will be two systematic reviews and a clinical trial that aims to investigate the effectiveness of a telerehabilitation program for patients with chronic musculoskeletal pain. However, throughout the project, there is no mention of the need to develop the telerehabilitation program and the information only appears when describing the activities of the fellow. I believe this information should be described in the research project as a step to be developed, specifying the content better and assessing whether it can be recommended for patients with chronic pain without first undergoing a thorough evaluation by the physiotherapist.

Another aspect is the very broad age group (1865 years). It is important to note that in Brazil from 60 years old onwards he is already considered elderly and therefore very different from a young man of 18 years old.

I also call attention to the language of the project submitted to FAPESP English and as it is a Brazilian development agency I suggest the Portuguese language.

Answer: Thank you for these important comments regarding the research project. The answer for each of them is described below for your consideration:

a) The development of the e-Rehab program is described in the research project in the "Statement of the problem" section as a tele-rehabilitation program based on a cognitive behavioral intervention program led by one of the associate researchers (Dr. Blake Dear) , called iCBT in addition to an exercise program. The iCBT project has shown promising results and we believe that the effects can be enhanced by the addition of an exercise component. Both components are described in more detail in Appendices 4 and 5. Regarding the initial assessment, all participants will be assessed for eligibility, medical history and factors that prevent the patient from performing the intervention. A thorough evaluation by the physiotherapist will not be necessary for this project, as it does not fit the intervention plan. The project aims to simplify health procedures for a possible global benefit and decrease waiting times in health centers. Thus, our goal is to verify the effectiveness of this proposed distance intervention, which also includes an initial assessment different from the conventional one. In addition, previous studies with remote interventions have also not used a thorough evaluation because the findings in the evaluation are unlikely to guide or alter this type of intervention, especially for chronic pain (in addition to not presenting explicit risks to patients).

b) Thanks for the suggestion. The age range of the project participants has been changed to 18-60 years, so we will not include patients considered elderly in Brazil.

c) FAPESP rules allow the submission of projects in English. As the main researcher of the project, I was careful to check with FAPESP if the project could be submitted in English, since half of the team of researchers are not Brazilian and participated in the development of the proposal. For this reason, we chose to present the project in English. In addition, I believe that choosing the English language for the project from the beginning should facilitate the further steps of the study (registration of the protocol, preparation of the manuscript and publication) as well as allowing all members of the research team to discuss the proposal.

Analyze the scientific basis and the methods used.

Methodology well described, just missing more information about the eRehab Program. It is important that the applicant checks with the Regional Council of Physiotherapy and Occupational Therapy in the CREFITO 3 region if the program does not violate the profession's ethical standards.

Answer: I believe that the question about the details of the e-Rehab program has been clarified in the previous answer, in the reformulated project and in the appendices. Regarding the second point of the referee, I

appreciate the comment and confirm that the present project is in accordance with the ethical and bioethical norms of physiotherapy in Brazil.

The project involves undergraduate or postgraduate students. Consider in this item only the active fellows with projects in progress (in a later item there is room for the analysis of new scholarships requested from FAPESP). The existence of scholarship holders supported by other sources is considered a relevant consideration by FAPESP.

[ ]Yes

[X] No

Answer: Nothing to declare.

Is the amount of resources requested justified in view of the importance of the intended scientific or technological contribution (quality and impact of the results)?

[ ]Yes

[X] No

Answer: I kindly ask the referee to reconsider this point, as the budget has been revised and updated. According to the previous clarifications, I believe that the project has great potential for impact in the area. If the e-Rehab program proves effective for patients with chronic pain, the implementation of this program has the enormous potential to benefit millions of people in the city of São Paulo (but also elsewhere in Brazil) who are waiting for treatment in the public health system. health care, in addition to allowing patients with truly emergency conditions to be seen in time.

The requested amounts are lower than the amounts used for similar projects around the world. Associate researcher Blake Dear, who has already been successful with over 16 million Australian dollars in tele-rehabilitation, will provide the basis for our project, which makes the requested amount much less than what would be needed if the project was to be elaborated from the beginning. However, it will still be necessary to create the delivery method for this project (example: website, videos, etc.). In addition, a large part of the budget is for scholarship costs, which will become the training of high quality human resources in Brazil.

Is the proposed deadline adequate for the development of the project?

[X] Yes

[ ] Not

Answer: Nothing to declare.

Analyze the scholarships requested as an integral part of the project (Scientific Initiation (IC), Technical Training (TT), Master's (MS), Direct Doctorate (DD), Pedagogical Improvement (EP) and Scientific Journalism (JC)). For the Young Researcher Project, FAPESP may grant, as part of the program budget, IC, TT, MS, DD, EP and JC scholarships, according to the rules described in: http://www.fapesp.br/bolsas/bolsasconcedidasemauxilios, by evaluating the project and the Activity Plan for each scholarship requested. If approved, the scholarship holder must be selected by the project applicant, according to FAPESP rules, available at the addresses mentioned in each case:

Analyze the adequacy of the quantity and Activity Plans proposed for Technical Training scholarship holders (http://www.fapesp.br/materia.php?data{id_materia/201=3171)

It is not described who will be the supervisor of the direct doctorate and I believe that, given the justifications, a TT scholarship is sufficient.

Answer: The doctoral student will be guided by the principal researcher of this proposal. This information was

added to the work plan for the doctoral scholarship. Regarding the technical training scholarships, we followed the opinion of the referee and withdrew the application for the TT-3 scholarship. The TT-4 scholarship refers to an information technology (IT) professional. As technology is the basis of the intervention to be tested in this project, we believe that having an IT professional is extremely important for the project's viability (for example, helping in the development of the program and correcting in the short term technological problems that may interfere with the system tele-rehabilitation). The coordinator of the PPG in physiotherapy at UNICID approves that a JP guides this student and, as a participant in the proposal, is committed to assisting me in orientation, if necessary.

Analyze the appropriateness of the quantity and Activity Plans proposed for Scientific Initiation fellows (http://www.fapesp.br/materia.php?data{id_materia/201=2889)

Not applicable

Answer: Nothing to declare.

Analyze the adequacy of the quantity and Activity Plans proposed for Master's scholarship holders (http://www.fapesp.br/8685)

Not applicable

Answer: Nothing to declare.

Analyze the appropriateness of the quantity and Activity Plans proposed for Direct Doctoral fellows (http://www.fapesp.br/materia/4789)

The activity plan is correct but the applicant is not accredited in a PostGraduation Program to guide a direct doctorate.

Answer: A letter from the coordinator of the institution linked to the project (Prof Dr Leonardo Costa) was attached to the project, guaranteeing that if this project is accepted by FAPESP, I will be accredited to the postgraduate program.

Analyze the appropriateness of the quantity and Activity Plans proposed for scholarships for Pedagogical Improvement (EP). (Http://www.fapesp.br/46)

Not applicable

Answer: Nothing to declare.

Analyze the adequacy of the quantity and Activity Plans proposed for Journalism scholarship holders

Scientific (JC). (http://www.fapesp.br/jornalismocientifico)

Not applicable

Answer: Nothing to declare.

Conclusion on the Analysis of the Research Project presented

[ ] Great.

[] Very good.

[] Very good, with some easily remedied deficiencies.

[X] Good.

[] Good with disabilities.

[ ] Regular.

[] With serious deficiencies.

Answer: Based on the clarifications above and changes in the proposal, I kindly ask the referee to review this criterion and I hope that the referee's doubts have been resolved.

Please review the PROPOSED BUDGET for:

Need for Equipment and Permanent Materials requested to carry out the project, also taking into account the infrastructure already available at the institution and the ability of the applicant and his team to use them.

Does permanent equipment and materials, national or imported, with a value greater than R $ 20,000.00 have your need justified in the proposal?

[X] Yes

[ ] Not

Answer: Nothing to declare (remembering that the amounts are less than R $ 20,000.00)

Opinion, in each case, on the validity of the justification presented in the item above

Justified

Answer: Nothing to declare.

Are there permanent equipment and materials for which FAPESP should or can request the establishment of a system of access to third parties not involved in this Project?

Not applicable

Answer: Nothing to declare.

Analyze the need for consumables requested for the project.

Not applicable

Answer: Nothing to declare.

Analyze the need and adequacy of Third Party Services for the realization of the project

Are Third Party Services only of a technical and occasional nature, as required by FAPESP rules?

The imported imported third party services do not apply as it is the disclosure on Facebook and Dropbox.

Answer: The budget has been revised and updated and these services have been withdrawn from the budget.

In any case, is it reasonable to expect that the requested service should be provided by the institution hosting the project, in return for support from FAPESP?

Can be provided by the host institution

Answer: Nothing to declare.

Suggest alternative values for the budget (Should be excluded from the budget: salaries of any kind, third-party services that are not of a technical and eventual nature, civil works, acquisition of

publications, trips except for field research, material and administrative services).

National permanent material.

It's suitable

Answer: Nothing to declare.

Imported permanent material.

Not applicable

Answer: Nothing to declare.

National consumption material.

Not applicable

Answer: Nothing to declare.

Imported consumables.

Not applicable

Answer: Nothing to declare.

Third party services in the country.

Some of the third party services in the country are not only of a technical nature, such as: printed matter, publication in the newspaper, SMS, monthly telephone line and these can be offered by the institution where the research will be carried out.

I also see some very high prices that I believe need more than a budget: video production, logo creation, website programming, etc., with a total value of R $ 199,109.88 reais.

Answer: The budget has been revised and updated. Disclosure, telephone and telephone line services were removed from the budget. The costs of third parties in relation to the development of the tele-rehabilitation system are extremely important for the success of this project. For this cost, we quote some communication companies in São Paulo and use the most affordable budget. However, we added three quotes from different companies to the project offering the cost of options for this service. The budget of Golombek, Pravy and Santa I / O was added.

Third party services abroad.

The imported imported third party services do not apply as it is publicized on Facebook and Dropbox since the survey will be conducted in Brazil.

Answer: The budget has been revised and updated. These services were removed from the budget, since the institution linked to the project must offer resources for the dissemination of the project and data storage.

Transport expenses in the country.

Not applicable

Answer: Nothing to declare.

Grand total national currency.

The total amount in national currency is quite high R $ 876,492.16 reais

Answer: I kindly ask the referee to reconsider this point, as the budget has been revised and updated.

Grand total foreign currency.

Value of $ 1,490.40 dollars

Answer: Nothing to declare.

Scholarships Based on the analysis carried out in the previous item, suggest the amount of IC, TT, MS, DD, EP and JC scholarships.

0 Scientific Initiation (IC) 0 Technical Training I (TTI) 0 Technical Training II (TTII) 1 Technical Training III (TTIII) 1 Technical Training IV (TTIV) 0 Technical Training IVa (TTIVa) 0 Technical Training V (TTV) 0 Master (MS) 1 Direct PhD (DD) 0 Pedagogical Improvement (EP) 0 Scientific Journalism

Answer: Nothing to declare.

Conclusion on the proposed budget

[] Suitable

[] May be Adequate if changes suggested above are made

[X] Inappropriate

Answer: As stated earlier, the budget has been revised and updated. I kindly ask the referee to reconsider this point and also consider that the higher cost of this budget is due to the creation of the tele-rehabilitation system for the study intervention. As this will be the first time that this system will be developed in Brazil for this condition, it is understood that the process must cost what is necessary to ensure that the study reports the real benefits. However, it is also understood that once this system is created and shows benefits (as it has been demonstrating in other countries), it may serve as a basis for other tele-rehabilitation programs in the future using the same structure and bringing even more benefits to the health system in Brazil. In addition, part of the intervention (iCBT) has already been created and will only be adapted and translated, which reduces costs that could be even higher. The researcher associated with this project Dr Blake Dear, creator of the iCBT program, developed this program with an initial budget of A $ 315 thousand, and a research budget for the development of other programs in the area worth A $ 16 million. Therefore, this is a great opportunity to establish tele-rehabilitation in Brazil at a viable cost. We believe that the project has great potential for impact in the area and we hope to have clarified the referee's doubts with the above clarifications. In addition, a large part of the budget is budgeted for grants, which will become the training of high quality human resources in Brazil.

NOTED DISABILITIES (If any item is checked, the reasons must be indicated in the corresponding table on the form)

About the Researcher's Academic Background:

[] Scientific or technological production that does not attest to significant performance of the research activity.

[] Insufficient experience in the research area in which the project is inserted, which may compromise its viability.

[] The scientific or technological production resulting from previous aid is unsatisfactory.

[] The applicant does not meet the requirements of the program rules.

Answer: Nothing to declare.

About the Institution and the Research Group:

[] The Institution's institutional and physical infrastructure is insufficient. [] Lack of institutional commitment to the success of the proposal. [] The proposal will not contribute to the nucleation of new research groups. [] The proposal will not contribute to the decentralization of the state research system. [] Center with a tradition of research in the area in which the project is inserted, with no exceptional circumstances in the light of the program rules.

Answer: Nothing to declare.

About the project:

[] Project with ill-defined, excessive or incongruous objectives.

[] Project with excessively limited objectives.

[] Unoriginal design.

[] Insignificant contribution to the area of knowledge.

[] Poor scientific foundation / inadequate methodology.

[] Feasibility of questionable execution.

[X] Excessive cost in view of the expected scientific or technological contribution or the probability of success of the project.

[] Activity plans for TT fellows are unsatisfactory.

[] The activity plans for IC fellows are unsatisfactory.

[] Activity plans for MS fellows are unsatisfactory.

[] Activity plans for DD fellows are unsatisfactory.

[] The activity plans for EP fellows are unsatisfactory.

[] Activity plans for JC fellows are unsatisfactory.

[X] The number of TT fellowships requested is inadequate.

[] The number of IC fellowships requested is inadequate.

[] The number of MS scholarship holders requested is inadequate.

[] The number of DD scholarship holders requested is inadequate.

[] The number of EP fellows requested is inadequate.

[] The number of JC fellows requested is inadequate.

[] Inadequate term.

Answer: As shown earlier, I would kindly ask the referee to reconsider this point in accordance with the budget modifications and updates. As stated earlier, we will be using part of an existing program and, therefore, the costs become more viable, enabling the creation of a first tele-rehabilitation system in Brazil. This project is promising and may be one of the greatest scientific advances in the Brazilian health system supported by FAPESP, and it may benefit millions of people in the future. According to the information previously clarified in relation to my school transcript and curriculum summary, I believe that I have full capacity to lead this project and guarantee a high quality of the study. In addition, the 3 associated researchers included in this proposal, have extensive experience with the indicated methodology, which increase the chances of success of this study and guarantee the viability. Prof Dr Leonardo Costa has already conducted several clinical trials in

his research group in Brazil. Dr Blake Dear has already managed over 18 million Australian dollars in research with tele-medicine and clinical trials. Professor Chris Maher is now ranked number 1 in low back pain research and number 4 in chronic pain according to expertscape.com, and has coordinated more than 15 clinical trials, the latest published in the New England Journal of Medicine . In addition, the number of TT fellowships requested was also changed in the new proposal in order to reduce the total costs of this project.

About the Proposed Budget:

[] Items insufficiently justified.

[X] Overestimated budget.

Answer: As stated earlier, the budget was revised and updated according to the suggestions of the referees. Therefore, I kindly ask the referee to reconsider this analysis. The budget of three different communication companies was included in the project.

Others (explain):

Conclusion

The applicant's scientific production is significant with 50 articles published in selective editorial policy journals, of which 28 were published in 2016. There are no published books or book chapters or other results. The research project is interesting but contains some inconsistencies. The Institution has a tradition in research. The project does not characterize a new line of research since several of the Institution's professors are already carrying out research on this topic. I suggest that the budget be revised as the figures are very high.

Answer: Thank you very much for taking the time to evaluate this proposal. I kindly ask that the referee reconsider his analyzes, based on the clarifications above, the modifications of the proposal and the new documents attached to the project.

Opinion 3rd Advisory

GENERAL ANALYSIS OF THE PROPOSAL FAPESP calls "Proposal" the set of four parts to be analyzed, composed of: 1. Academic History of the Researcher (a) Responsible; 2. Research Project; 3. Conditions offered by the Institution where the project will be developed; and, 4. Budget. Please complete this item after completing the other items on this form.

Academic background of the researcher: Excellent. Research project: Relevant and relevant to the area of knowledge, but there are methodological errors that must be corrected so that it does not compromise the feasibility and results of the research. Conditions offered by the institution: Adequate. Budget: The national third party service item should be reviewed.

Answer: Thank you for evaluating this proposal. The points mentioned related to the study methods and the budget are explained point by point below.

Please review the RESEARCHER'S RESPONSIBLE ACADEMIC HISTORY of the Project considering the items below:

Analyze the quality and regularity of scientific and / or technological production. Important elements for this analysis are: list of publications in journals with selective editorial policy; books or book chapters; patents in which he appears as an inventor; other intellectual property instruments; research results effectively transferred and adopted by companies or the government; and other information that may be relevant.

The researcher has excellent scientific production with articles published in journals of selective editorial policy and an excellent impact factor.

There are 40 articles and two book chapters

Answer: Nothing to declare.

Analyze the experience demonstrated in research projects related to the theme of the proposed project.

He has experience in systematic review projects and clinical trials.

Answer: Nothing to declare.

Analyze the candidate's international research experience (short and long internships, participation in collaborations), considering the quality of the published results.

The researcher completed his doctoral thesis in Australia and produced articles of clinical relevance and published in journals with a high impact factor.

Answer: Nothing to declare.

When appropriate, analyze the results obtained by the applicant with previous financing from FAPESP or other agencies, including scholarships.

Nothing to declare.

Answer: Nothing to declare.

Analyze other items that make up the curriculum summary.

According to the rules of the program, the applicant must be a researcher still in the stage of professional affirmation, but already with a very good production for the stage of his career. In particular, the candidate must be fully capable of coordinating research projects independently and implementing new lines of research. Does the applicant fulfill these requirements?

[X] Yes

[ ] Not

Justify:

Answer: Nothing to declare.

Conclusion on the analysis of the Academic History of the Researcher Responsible for the Project.

[X] Excellent

[ ] Very good

[ ]Good

[ ] Regular

[] With serious deficiencies

Answer: Nothing to declare.

Please review the INSTITUTIONAL CONDITIONS in which the proposal is inserted, following the script below.

Analyze the institution's research tradition. Is the project characterized as a new line of research at the institution with a consolidated tradition in research?

It is not a new line of research at the institution, but the institution has an emerging tradition of research on systematic review and low back pain.

Answer: My professional objective for the coming years is to develop a line of research in remote interventions for different health conditions. Although there are studies of low back pain in the institution to which this project is linked, the line of research with remote interventions in chronic pain is not part of any research of the institution and, in addition, there seems to be no researchers in Brazil with a line research related to remote interventions. I believe that this line of research in remote interventions is extremely important for our country, especially in the city of São Paulo, where the health service has a very high demand. This type of intervention is growing exponentially in the world. According to a report in the Wall Street Journal (USA), more than 15 million Americans received some type of remote intervention through technologies in 2016 and this number is expected to increase by 30% in 2017. According to health technology expert Larry Jones, he estimates It is noted that in 2018 more than 90% of large American companies (+1,000 employees) are expected to offer tele-medicine services (distance medical services) to their employees. In addition, the American College of Physicians (ACP), in its latest recommendation on the subject, strongly recommends the use of tele-medicine in clinical practice to amplify access to health and decrease health costs. So I believe that this line of research is very promising to be developed in Brazil and will add to the institution in which this project is linked.

The proposed project focuses on chronic pain due to my experience on the subject and the experience of the associate researchers. As my line of research consolidates in the future, I intend to continue with other chronic conditions that need attention (for example: diabetes and fibromyalgia). I currently have collaboration with the "Center for Pain, Health and Lifestyle - www.centrephl.org", a group located at the University of Sydney and at the University of Newcastle, where I am having the opportunity to work on projects with remote interventions for various conditions of health, such as a systematic review I'm leading on smoking control interventions on the impact of chronic pain, which includes distance interventions. There are professors at the institution linked to this project who study low back pain and neck pain, which will contribute to the development and viability of the line of research that I intend to work with this project.

References:

1. Link to article in the Wall St Journal: https://www.wsj.com/articles/how-telemedicine-is-transforming-health-care-1466993402?mod=itp&mod=djemITP_h

2. Link to Larry Jones article: https://www.linkedin.com/pulse/telehealth-living-up-promise-larry-jones

3. Daniel H, Sulmasy LS, for the ACP Health and Public Policy Committee. Policy recommendations to guide the use of telemedicine in primary care settings: an American College of Physicians position paper. Ann Intern Med 2015; published online 8 Sep, doi: 10.7326 / M15-0498.

4. Stevens ML, Lin CC, Hancock MJ, et al. TOPS: Trial Of Prevention Strategies for low back pain in patients recently recovered from low back pain - study rationale and protocol. BMJ Open 2016; 6: e011492. doi: 10.1136 / bmjopen-2016-011492.

Analyze the adequacy of the institutional and physical infrastructure offered by the Institution in which the project will be developed and the institutional commitment to the proposal, according to the program rules.

The institution has an adequate structure for conducting the research

Answer: Nothing to declare.

Analyze the effects that support for the proposal may have on the Institution. Note that: 1) The purpose of the Young Researchers in Emerging Centers Program is to enable the creation of adequate job opportunities for young researchers or groups of young researchers with great potential, preferably in emerging research centers, favoring the nucleation of new groups and the decentralization of the state research system. Candidates who intend to create a new line of research in an institution with a consolidated research tradition may also enroll in the program; 2) in the case of an institution with a tradition of research, the proposal will be approved only if: a) It is a candidate with exceptional

performance for the stage of his career; b) Special circumstances are identified (for example, it is a proposal to create a new research group in the unit or department) that may justify the support of the proposal in this program.

The institution is an emerging research center and the responsible researcher has great potential in the elaboration and execution of the research.

Answer: Nothing to declare.

Conclusion on the Institutional Conditions where the Project will be carried out

[ ] Great

[X] Very good

[ ]Good

[ ] Regular

[] With serious deficiencies

Answer: Nothing to declare.

Please review the RESEARCH PROJECT following the script below.

Analyze the definition, relevance, originality of the objectives and the importance of the intended contribution to the area of knowledge in which the proposed project is inserted.

The project is original and has well-defined objectives and a significant scientific contribution to the area of knowledge.

Answer: Nothing to declare.

Analyze the scientific basis and the methods used.

Scientific background: References on chronic pain are more than 10 years old, and most of them in low impact magazines. Chronic pain data should be updated.

The project describes that it will recruit people with chronic pain with at least 3 according to the numerical scale for pain, but there is no maximum.

It is not described whether patients will be able to use medication to control pain, anxiety and depression. The use of medication can be a confusing factor and can lead to errors in the interpretation of results. People who have chronic, according to the literature, usually evolve with mood swings, anxiety and depression. Therefore, they can use medications to control the mental health condition that can interfere with pain. The researcher must have control over the use of medication, even though it may occur in both groups.

Will patients with pain 10, according to the numerical scale, be able to perform the intervention?

What do the authors call serious illnesses? (Tumor, inflammatory, fracture and infection). Rheumatoid arthritis can trigger significant pain, or, if controlled, it does not present as much pain and is an inflammatory disease. Will patients with this disease be excluded?

Define serious heart and metabolic disease. What diseases will be excluded?

"The study will be designed to detect a difference of 1 point in a 0 to 10 Pain Numerical Rating Scale, estimated standard deviation of 1.75 points, statistical power of 90%, alpha level of 5%, and followup loss of 15% . " Why was the difference of 1 point on the numerical pain scale (END) used, given that the minimum clinically important improvement for pain assessed by END is two points? One point does not

represent clinically important improvement.

Researchers will offer a DVD to patients who do not have access to the internet or computer, but what if patients do not have a DVD player or computer? Will equipment be offered to patients?

In the introduction, the researchers state that telerehabilitation reduces treatment costs. I understand that it is not the objective of the research to evaluate the treatment costs, but it must be demonstrated that the cost is, in fact, lower in relation to the conventional treatment.

Answer: Thanks for the suggestions, comments and questions. The answer for each point is described below:

a) References on chronic pain were reviewed and updated in the project. However, unfortunately, references on epidemiological data on chronic pain in Brazil are not updated.

b) There is no maximum pain level for the inclusion of study participants. We believe that patients with high pain levels should benefit from the intervention, as well as patients with moderate pain (for example, between 3 and 6 points / 10). However, the progression levels of the intervention may be altered according to the individual's ability to perform the suggested intervention (that is, if a patient is unable to perform all exercises in week 1 of the intervention due to pain, he can repeat this program instead of progressing to week 2). In addition, due to the characteristic of chronic pain, we understand that patients with this condition do not tend to stagnate at level 10 for a long time, allowing them to carry out the proposed interventions in a short period.

c) Thanks for this comment. Patients with rheumatoid arthritis will not be included, as it is an autoimmune inflammatory disease. We added this information in the new version of the project.

d) Thanks for this comment. Some examples of serious cardiac or metabolic diseases that will prevent participants from being included in the study (example: coronary artery disease, heart failure and decompensated diabetes) have been added to the new version of the project.

e) Thanks for the comment. After a careful review of this point with the researchers involved in the project, we decided to recalculate the sample size based on a recent clinical trial published in the New England Journal of Medicine (Mathieson 2017). Therefore, we considered a difference between the groups of 1.5 points in the VAS, which provides a representative sample for the study (160 subjects). Increasing the difference between groups for the sample calculation (for example, 2 points), would drastically decrease the sample of this study, which has been a limiting factor for the statistical power of clinical trials in the area.

The full description of the sample calculation has been added to the section "3.8.1 Sample Size Calculation", and is described below:

"We determined that a minimum of 160 individuals (80 per group) would be required to provide the trial with 90% power to detect a between-group difference of 1.5 point in a 0 to 10 Pain Numerical Rating Scale, with a standard deviation of 2.75 points and two-sided alpha level of 0.05. The estimated sample size would also allow for a withdrawal rate of 15%. "

reference:

Mathieson S, Maher CG, McLachlan AJ. Trial of Pregabalin for Acute and Chronic Sciatica. New Engl J Med. 2017; 376 (12): 1111-1120.

f) Internet access in Brazil grows every year, but it is still considered low in relation to other countries. For example, in 2015 it was registered that 60% of households have internet access in Brazil compared to 85% in the United States in the same year. Therefore, in this study we decided to offer the option of the content of the e-Rehab program on an interactive DVD for patients without access or with little access to the internet.

Unfortunately, patients who do not have a DVD player (or computer with a DVD input) OR, who do not have access to the Internet cannot be included in the study, since it would be impracticable for the project to provide a DVD player or computer for all participants. This was added to the project, along with the exclusion criteria.

g) Thanks for the comment. Although we do not have the primary objective of assessing the costs of this intervention, this information has been added to the project for reporting. Results from a 2003 study suggested that among patients who do not participate in the tele-medicine service, 94% travel more than 110km for medical appointments, 84% miss a day at work to go to a medical appointment and 74% spend between $ 75- $ 150 in additional consultation costs (Bynum 2003). The Johns Hopkins University remote program called "hospital at home" reduced service costs by 19% compared to conventional services (Cryer 2012). In addition, a recent study found that the tele-rehabilitation program based on cognitive behavioral therapy was more cost-effective than cognitive behavioral therapy performed in the office (de Boer 2014). This information was added to the revised project.

References:

1. Cryer L, Shannon SB, van Amsterdam M, Leff B. Costs For 'Hospital At Home' Patients Were 19 Percent Lower, With Equal Or Better Outcomes Compared To Similar Inpatients. Health Affairs. 2012; 31 (6): 1237-1243. doi: 10.1377 / hlthaff.2011.1132.

2. Bynum AB, Irwin CA, Cranford CO, Denny GS. The impact of telemedicine on patients' cost savings: some preliminary findings. Telemed J E Health. 2003; 9 (4): 361-7.

3. de Boer MJ, Versteegen GJ, Vermeulen KM, et al. THE

Is the proposed deadline adequate for the development of the project?

[X] Yes

[ ] Not

Answer: Nothing to declare.

Conclusion on the Analysis of the Research Project presented

[ ] Great.

[] Very good.

[] Very good, with some easily remedied deficiencies.

[ ]Good.

[X] Good with disabilities.

[ ] Regular.

[] With serious deficiencies.

Answer: According to the changes, clarifications and revisions of the proposal, I kindly ask the referee to reconsider this analysis and I believe that the referee's doubts about the project have been resolved.

Please review the PROPOSED BUDGET for:

Need for Equipment and Permanent Materials requested to carry out the project, also taking into account the infrastructure already available at the institution and the ability of the applicant and his team to use them.

Does permanent equipment and materials, national or imported, with a value greater than R $ 20,000.00 have your need justified in the proposal?

[X] Yes

[ ] Not

Grand total national currency.

R $ 902,539.94.

Grand total foreign currency.

R $ 1,490.40

Scholarships Based on the analysis carried out in the previous item, suggest the number of IC, TT, MS, DD, EP and JC scholarships.

0 Scientific Initiation (IC) 0 Technical Training I (TTI) 0 Technical Training II (TTII) 1 Technical Training III (TTIII) 1 Technical Training IV (TTIV) 0 Technical Training IVa (TTIVa) 0 Technical Training V (TTV) 0 Master (MS) 0 Direct Doctorate (DD) 0 Pedagogical Improvement (EP) 0 Scientific Journalism

Answer: Nothing to declare.

Conclusion on the proposed budget

[] Suitable

[X] May prove to be suitable if changes suggested above are made

[] Inappropriate

Answer: Changes were made to the budget as suggested by the referee.

DEFICIÊNCIAS NOTADAS (Se algum item estiver assinalado, as razões devem estar indicadas no quadro correspondente do formulário)

About the project:

[] Project with ill-defined, excessive or incongruous objectives.

[] Project with excessively limited objectives.

[] Unoriginal design.

[] Insignificant contribution to the area of knowledge.

[X] Poor scientific foundation / inadequate methodology.

[] Feasibility of questionable execution.

[X] Excessive cost in view of the expected scientific or technological contribution or the probability of success of the project.

[] Activity plans for TT fellows are unsatisfactory.

[] The activity plans for IC fellows are unsatisfactory.

[] Activity plans for MS fellows are unsatisfactory.

[] Activity plans for DD fellows are unsatisfactory.

[] The activity plans for EP fellows are unsatisfactory.

[] Activity plans for JC fellows are unsatisfactory.

[] The number of TT fellowships requested is inadequate.

[] The number of IC fellowships requested is inadequate.

[] The number of MS scholarship holders requested is inadequate.

[] The number of DD scholarship holders requested is inadequate.

[] The number of EP fellows requested is inadequate.

[] The number of JC fellows requested is inadequate.

[] Inadequate term.

Answer: As for the scientific basis, I believe that I have the capacity to lead this project and guarantee a high probability of success for the study. In addition, the 3 associated researchers have extensive experience with the proposed methodology, which increases the chances of success of this study. Prof Dr Leonardo Costa has already conducted several clinical trials in his research group in Brazil. Dr Blake Dear has already managed over 18 million Australian dollars in research with tele-medicine and clinical trials. Professor Chris Maher is now ranked number 1 in low back pain research and number 4 in chronic pain according to expertscape.com, and has coordinated more than 15 clinical trials.

I believe that the answers to the questions above, as well as the changes and updates in the project, have solved any doubts of the referee in relation to the methodology and quality of the study. The study design is a randomized controlled study to test the effectiveness of a tele-rehabilitation program, which we believe is the best study design for this purpose. Among the associate respondents, three of them are researchers with extensive experience in clinical trials who have reviewed the study plan and who have published their clinical trials in renowned scientific journals such as the New England Journal of Medicine, The Lancet and Pain.

I kindly ask that the referee also reconsider the point related to the budget, since the budget has been modified and updated. As stated earlier, we will be using part of an existing program and, therefore, the costs become more viable, enabling the creation of a first tele-rehabilitation system in Brazil. This project is promising and may be one of the greatest scientific advances in the Brazilian health system supported by FAPESP, and it may benefit millions of people in the future. As this will be the first time that this system will be developed in Brazil for this condition, it is understood that the process must cost what is necessary to ensure that the study reports the real benefits. However, it is also understood that once this system is created and shows benefits (as it has been demonstrating in other countries), it may serve as a basis for other tele-rehabilitation programs in the future using the same structure and bringing even more benefits to the health system in Brazil. In addition, part of the intervention (iCBT) has already been created and will only be adapted and translated, which reduces costs that could be much higher. The researcher associated with this project Dr Blake Dear, creator of the iCBT program, developed this program with an initial budget of A $ 315 thousand, and a research budget for the development of other programs in the area worth A $ 16 million. Therefore, this is a great opportunity to establish tele-rehabilitation in Brazil at a viable cost. We believe that the project has great potential for impact in the area and we hope to have clarified the referee's doubts with the above clarifications.

About the Proposed Budget:

[X] Items insufficiently justified.

[] Overestimated budget.

Answer: In addition to budget modifications and updates, we added three quotes for third party items in Brazil. I therefore ask the referee to reconsider this analysis.

Others (explain):

Conclusion

The project can be corrected and improved and the national third party service item can be better justified and the inclusion of another budget.

Answer: Thank you for the opinion and the evaluation of this proposal. I believe that the answers above and the new attached documents may clarify the points raised by the referee.

Coordination Recommendation

There are criticisms of the rationale and methods of the project. Excessive cost due to the scientific contribution of the project.

Answer: According to the clarifications above, I believe I have solved these points mentioned in relation to the rationale, methods and budget.

3- ACRESCENTE COMENTÁRIOS QUE CONSIDERE IMPORTANTES PARA A ANÁLISE DA RECONSIDERAÇÃO

(É oportuno lembrar que, caso, por qualquer razão, entenda-se ser necessário e cabível contestar a qualidade do parecer de assessoria ou a própria adequação da assessoria, isso deverá ser feito em carta à parte, dirigida ao Diretor Científico, justificando a solicitação de que seja ouvido novo assessor)

Nothing to declare

4- place, date

Sydney, 20 August 2017
